# A prognostic signature model for unveiling tumor progression in lung adenocarcinoma

**DOI:** 10.3389/fonc.2022.1019442

**Published:** 2022-11-01

**Authors:** Zijian Li, Tao Zeng, Chong Zhou, Yan Chen, Wu Yin

**Affiliations:** ^1^ State Key Lab of Pharmaceutical Biotechnology, College of Life Sciences, Nanjing University, Nanjing, Jiangsu, China; ^2^ Department of Chinese Medicine, Jiangsu Cancer Hospital & Jiangsu Institute of Cancer Research & The Affiliated Cancer Hospital of Nanjing Medical University, Nanjing, Jiangsu, China

**Keywords:** lung adenocarcinoma, tumor microenvironment, single-cell RNA sequence, risk score, tumor progress

## Abstract

A more accurate prognosis is important for clinical treatment of lung adenocarcinoma. However, due to the limitation of sample and technical bias, most prognostic signatures lacked reproducibility, and few were applied to clinical practice. In addition, understanding the molecular driving mechanism is indispensable for developing more promising therapies for lung adenocarcinoma. Here, we built an unbiased prognostic significance model to perform an integrative analysis, including differentially expressed genes and clinical data with lung adenocarcinoma patients from TCGA. Multivariable Cox proportional hazards model with the Lasso penalty and 10-fold cross-validate were used to identify the best gene signature. We generated a 17-gene signature for prognostic risk prediction based on the overall survival time of lung adenocarcinoma patients. To further test the model’s predictive ability, we have applied an independent GEO database to verify the predictive ability of prognostic signature. The model can more objectively describe several biological processes related to tumors and reveal important molecular mechanisms in tumor development by GO and KEGG analysis. Furthermore, differential expression analysis by GSEA revealed that tumor microenvironments such as ER stress, exosome, and immune microenvironment were enriched. Using single-cell RNA sequence technology, we found that risk score was positively correlated with lung adenocarcinoma marker genes and copy number variation but negatively correlated with lung epithelial marker genes. High-risk cell populations with the model had stronger cancer stemness and tumor-related pathway activation. As we expected, the risk score was in accordance with the malignancy of each cluster from tumor progression. In conclusion, the risking model established in this study is more reliable than others in evaluating the prognosis of LUAD patients.

## Introduction

Lung adenocarcinoma (LUAD) is the most common subtype of lung cancer and a significant cause of cancer-related death worldwide (1, 2). Most LUAD patients are diagnosed in advanced or metastatic stages, which is the primary cause of mortality in lung cancer (3). However, LUAD patients’ prognosis is far from satisfying, and its associated microenvironments remain poorly understood (1). Even stage I lung cancer has a poor prognosis with 5-year overall survival (OS) after surgical resection (4), revealing the need for treatment escalation, such as adjuvant therapy. Notably, LUAD is a complex disease involving multiple pathways in pathogenesis. Thus, an in-depth understanding of the driven molecular mechanisms of LUAD is indispensable for developing more promising therapies.

Investigators have continued to seek prognostic signature that are predictive of survival benefit, as it is the basis for developing personalized approaches to improve the survival of early-stage lung cancer patients (5, 6). Many studies have proposed genomic signatures for risk score and survival prediction in lung cancer patients (7–9). However, most prognostic signatures lacked reproducibility due to problematic issues such as limited sample size, individual heterogeneity, and technical bias, few prognostic signatures were applied to routine clinical practice (6). The study built a significant prognostic model to perform an integrative analysis including differentially expressed genes (DEGs) and clinical data with lung adenocarcinoma patients from The Cancer Genome Atlas (TCGA) Program. Multivariable Cox proportional hazards model with the Lasso penalty and ten-fold cross-validation were used to identify the best gene signatures among different gene categories. We generated a 17-gene signature for prognostic risk prediction based on OS time with LUAD patients.

Efforts for understanding lung cancer progression have primarily focused on the profiling of cancer cells with genetic aberrations (10, 11). However, progression also can be influenced by complex and dynamic features from the tumor surroundings (12). For learning more about tumor progression, genetically engineered mice (GEM) with somatic mutation of Kras-G12D with or without TP53 deletion in alveolar type 2 cells (termed ‘K’ mice and ‘KP’ mice) can spontaneously suffer lung adenoma (13, 14), and the adenoma in ‘KP’ mice can progress into advanced LUAD over more than 12 weeks (15–17). This technique can control the tumor progress with increased accuracy. Single-cell RNA sequence (scRNA-seq) can offer more details about tumor, while bulk RNA sequence can only offer an overview description. Additionally, it can be a powerful tool to characterize each cell in a tumor, which could help us understand more about tumor characteristics (18–20). To validate the survival, scRNA-seq and GEM were used to identify more details regarding tumor progression through the prognostic signature.

## Material and methods

### Data collection

RNA sequence and clinical data with lung adenocarcinoma patients from TCGA were downloaded and prepared using the R ‘TCGAbiolinks’ package (21). The gene expression profiles include 520 primary tumor samples, which could correspond with clinical data and 59 normal tissue. Gene expression omnibus (GEO) database including GSE43458 (110 samples), GSE10072 (107 samples), GSE32863 (116 samples), GSE31210 (246 samples) and GSE50081 (181 samples) were downloaded by ‘GEOquery’ R package (22). Probs in GSE43458 (110 samples), GSE10072 (107 samples) and GSE32863 (116 samples) were annotated by ‘hgu133a.db’ and ‘annotate’ R packages; Probs in GSE31210 and GSE50081 were annotated by ‘hgu133plus2.db’ and ‘annotate’ R package (23). When a gene was mapped to different probes, the genic expression value was calculated by the average expression value. Furthermore, gene expression data from the GEO (https://www.ncbi.nlm.nih.gov/geo/) and GDC databases (https://cancergenome.nih.gov/) were z-score transformed for survival analysis. The clinical features of the TCGA and GEO patients is shown in **Table S1** and **Table S2**.

### Construction prognostic signature

The ‘limma’ R package performed differential expression analysis on primary tumor and normal tissues (24). P-values and fold changes were controlled for false discovery rate (FDR< 0.05 and |logFC| > 0.5). The ‘survival’ R package performed univariate Cox analyses of OS to identify the prognostic signature (25). The “Coxph” function was used to build a Univariate Cox model and calculate the p-value and C-index (consistency index). In addition, the “Cox.zph” function was used to test the proportional hazards assumption for a Cox regression model fit. (p< 0.05 and C-index > 0.58 and p-value of Proportional Hazards Assumption larger than 0.4).

Multivariable Cox regression analysis was performed to analyze the overall probability of survival. Lasso regression was performed by the ‘glmnet’ R package to reduce the number of genes, and a 10-fold cross-validation was performed to set proper lambda, and finally, 17 genes were left (26).


$$RiskScore=\sum_{i\in RiskGenes}w_{i}*mRNA_{i}$$

*w* represents the Lasso coefficient index of risk genes, *mRNA* represent gene expression, and the gene expression level of each gene, respectively.

The differential between high risk (HR > 1) and low risk (LR< 1) was confirmed by Kaplan-Meier method (log-rank test). The ‘timeROC’ R package performed a receiver operating characteristic curve (ROC) analysis to assess the predictive efficiency of the prognostic signature (27). C-index was used to evaluate the model’s predictive ability. Multivariable Cox regression was used to integrate different predictive factors (upper and lower limits of 95% confidence interval). The GEO database, including GSE31210 and GSE50081, validated the model. C20orf197 in GSE31210 and GSE50081 is missing, and the expression of C20orf197 in two databases was assigned 0.

### Function and enrichment analysis

Endoplasmic reticulum (ER) stress-related genes were collected from ‘Msigdb’ (28), and exosome-related genes were collected from ExoCarta (29, 30). The ‘GSVA’ R package was applied to perform a single-sample gene set enrichment analysis (ssGSEA) to quantify the ssGSEA score of immune signatures and endoplasmic reticulum stress and exosome-related genes (31). Differential genes in LUAD samples ad. pavlue< 0.05 and logFC > 0.5 or logFC< -0.5. Gene Ontology (GO) and Kyoto Encyclopedia of Genes and Genomes (KEGG) enrichment analyses were performed by the ‘clusterProfiler’ R package. Gene sets were collected from ‘Msigdb’ based on the DEGs, which was done by the ‘limma’ R package between high-risk and low-risk groups (28, 32). To estimate the activation of hallmark pathways, the GSEA analysis was applied with standard settings (32). To analyze the tumor immune microenvironment of the LUAD samples, the ‘GSVA’ R package was used to perform ssGSEA to quantify the ssGSEA score of 29 immune cell-related gene sets (33, 34). The Wilcox test tested the differential expression analysis of immune-related signatures, and the correlation between risk score and immune signatures was performed by Spearman correlation analysis. Differentially expressed genes about immune signatures were done by ‘limma’ R package (P.Value< 0.005). The same methods are performed in ER stress and exosome analysis.

### Single-cell analysis

Single-cell RNA sequence data (after depth normalization) was downloaded from GEO database (GSE154989). Seurat v4.0.4 was used for single-cell analysis (35–38). The count for the genes in each cell was log normalized, and the ScaleData function was used for scaling. SCTransform function was used for correcting different animals and plates, and the top 40 principal components were used to construct the SNN graph and embedding. FindClusters function was used for clustering cells at 0.5 resolution. AUCell v1.15.0 was used for scoring the ER stress and exosome gene sets in scRNA-seq (39). InferCNV v1.9.1 was used for the copy number variation (CNV) assignment with default parameters (40). Raw CNV score for each cell was collected from ‘infercnv.observations.txt’ file and transformed into 3-level scoring (0.7~1.3 assign 0; 0~0.3 or larger than 1.5 assigns 2; 0.3 ~ 0.7 or 1.3~1.5 assigns 1). Cluster 11, the earliest cell type T, was considered as the control and had no CNV. The CNV score for each cell was the sum of every gene’s CNV Score.

For estimating the trajectory of scRNA-seq, Monocle v2.21.1 was used to estimate and order cells in pseudotime along a trajectory (41). Several ablation experiments were done to select proper genes to estimate pseudotime. High variable genes calculated by Monocle and stemness genes collected from ‘Msigdb’ were used to estimate pseudotime. Cluster 11, which appeared in the early stage of LUAD, was set to the original point of pseudotime. Monocle V2 was used to obtain highly variable genes, DDRtree was used to establish the minimum spanning tree after dimensionality reduction.

### Statistical analysis

Multivariable and univariate Cox regression were used to analyze the probability of OS, and KM method was used to test the difference between high-risk and low-risk patients. Chi-squared test tested differences between risk scores and clinical information. ER stress scores, exosome scores, and immune signatures between the two groups were examined using Wilcox test and Spearman correlation analysis. Statistical analyses were performed using R v.4.1.1. The detailed analysis methods in the website (https://github.com/ZengTaox/xlw/blob/main/upload.R).

## Results

### DEGs identification and construction of a prognostic model in TCGA cohort

Five hundred ninety-two LUAD and adjacent non-tumorous samples from the TCGA database were included in DEGs and prognostic genes for OS. Meanwhile, 520 LUAD samples from the TCGA database and 427 patients from two GEO cohorts were incorporated into the following study about prognostic model.

**Figure 1:**
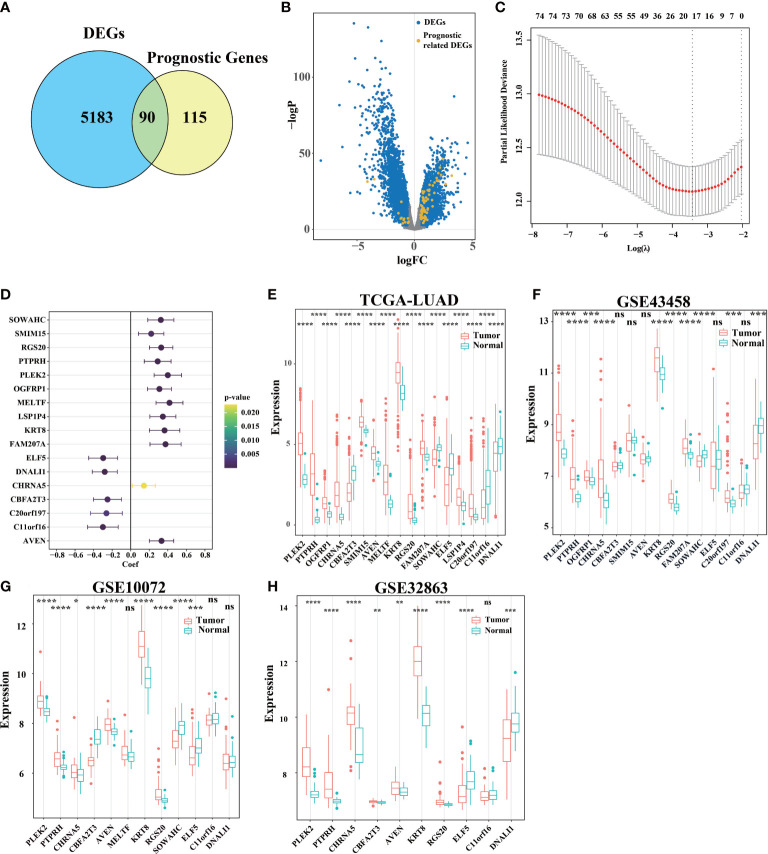
Identification of signature genes and establishment of a survival model. **(A)** A total of 5273 DEGs with adj. pvalue< 0.05 and logFC > 0.5 or logFC< -0.5 were selected by limma. 205 genes related to survival were found by stationarity test and univariate Cox regression analysis. Of these, 90 genes were associated with both survival and differential between the cancer and para-cancer groups. **(B)** Use volcano map to describe the distribution of DEGs in all genes. **(C)** LASSO Cox regression model was used to establish a multivariable model, and 10-fold cross validation was used to calculate the total deviation corresponding to differe nt penalty coefficients. **(D)** Univariate Cox regression test **(C)** The influence of the gene on survival (upper and lower 95% confidence intervals, color significance). **(E)** Analysis of gene expression differences between primary cancer (n = 533) and adjacent tissue (n = 59) in TCGA-LUAD. **(F–H)** DEGs in tumor tissue and para-cancer tissue in GEO databases (ns was non-significant, *P< 0.05, **P< 0.01, ***P< 0.001, ****P< 0.0001).

Firstly, compared tumor and adjacent non-tumorous tissues from TCGA and get DEGs. Secondly, we use univariate Cox regression analysis in tumor tissue from TCGA to get prognostic genes (p< 0.05), 90 of 205 prognostic genes were DEGs (**Figures 1A, B**). To further construct a risk scoring model for predicting possibility of OS in LUAD patients, LASSO Cox regression was used to build a prognostic model, which included *PLEK2*, *PTPRH*, *OGFRP1*, *CHRNA5*, *CBFA2T3*, *SMIM15*, *AVEN*, *MELTF*, *KRT8*, *RGS20*, *FAM207A*, *SOWAHC*, *ELF5*, *LSP1P4*, *C20orf197*, *C11orf16* and *DNALI1* (**Figure 1C**). The univariate Cox regression analysis suggests that these genes can be used as prognostic genes (**Figure 1D**), and can also be used to distinguish tumor from adjacent tissues. In addition, we evaluated three extra GEO cohorts (**Figures 1F–H**), the prognostic genes in the above GEO databases are consistent with TCGA databases (**Figure 1E**).

### Predictive performance and prognostic value of the model

**Figure 2:**
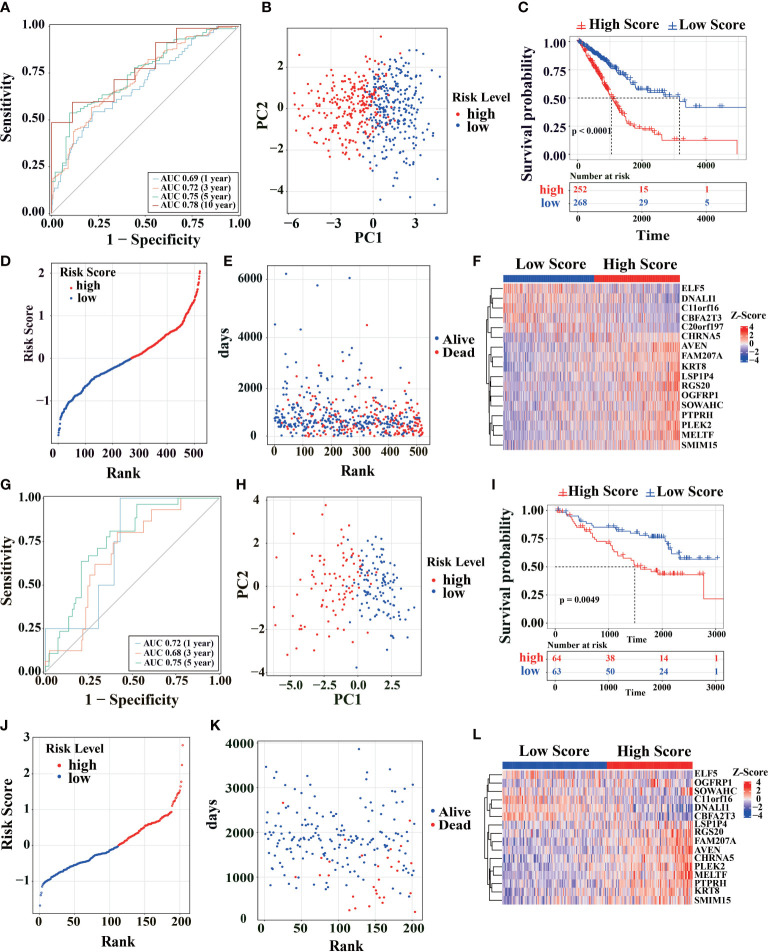
Survival analysis of the model in TCGA cohort. **(A)** Using TCGA-LUAD primary cancer samples and corresponding clinical records (n = 520) to calculate the AUC of time-dependent ROC curves of the risk score at 1, 3, 5 and 10 years. **(B)** PCA analysis was performed using the genes in **Figure 1C** of the primary LUAD sample in TCGA cohort. **(C)** The survival rates of LUAD patients were tested to test by KM survival analysis in the TCGA database. **(D)** Distribution map of different survival risks of LUAD patients in TCGA database. **(E)** Time distribution of LUAD patients with different survival risks and death in TCGA database (ordinate: follow-up time, abscissa: risk ranking). **(F)** Gene expression trends in **Figure 1C** of LUAD patients in TCGA database (top note: blue: low-risk, red: high-risk; Heat map below: Red: high expression, blue: low expression). **(G-L)** Patients from the GEO cohort (GSE31210) were analyzed similarly to the above analysis in TCGA cohort.

For predictive performance of the 17-gene prognostic model in TCGA cohort, the area under the curve (AUC) in the time-dependent ROC analysis reached 0.69, 0.72, 0.75, and 0.78 (**Figure 2A**), indicating excellent specificity and sensitivity of the risk score for predicting OS. According to the median value of risk score, the patients were divided into high-risk and low-risk groups. PCA analysis confirmed that patients in two groups were stratified into two directions (**Figure 2B**). KM analysis indicated that worse prognosis and significantly poorer OS were detected in high-risk patients (two-stage test P< 0.0001, log-rank test P< 0.001, **Figure 2C**). Compared with low-risk group, the patients had a higher proportion of death and shorter survival time in high-risk group (**Figures 2D, E**). *ELF5*, *DNALI1*, *C11orf16*, *CBFA2T3*, and *C20orf197* had higher expression levels in low-risk group, while the other genes in the prognostic signature had higher expression levels in high-risk group (**Figure 2F**).

To further estimate the model’s generalization performance, we have validated the predictive ability of prognostic signatures in the GEO database (GSE31210, **Figures 2G–L**; GSE50081, **Figures S1A–F**). In GSE31210 cohort, the AUC was 0.72 at 1 year, 0.68 at 3 years, and 0.75 at 5 years (**Figure 2G**). Similar to TCGA, the patients from GSE31210 cohort were divided into different directions by PCA analysis (**Figure 2H**). KM survival analysis indicated that high-risk patients had a higher proportion of death (log-rank test P< 0.0049, **Figure 2I**) and shorter survival time (**Figures 2J, K**). The ELF5, DNALI1, C11orf16, and CBFA2T3 genes also had higher expression levels in the low-risk group in GSE31210 cohort (**Figure 2L**). The same results are shown in GSE50081 cohort (**Figures S1A–F**). These similar results show that the signature has good generalization performance and can potentially predict prognosis for LUAD patients.

### The signature score as an independent prognostic factor in clinical features

**Figure 3:**
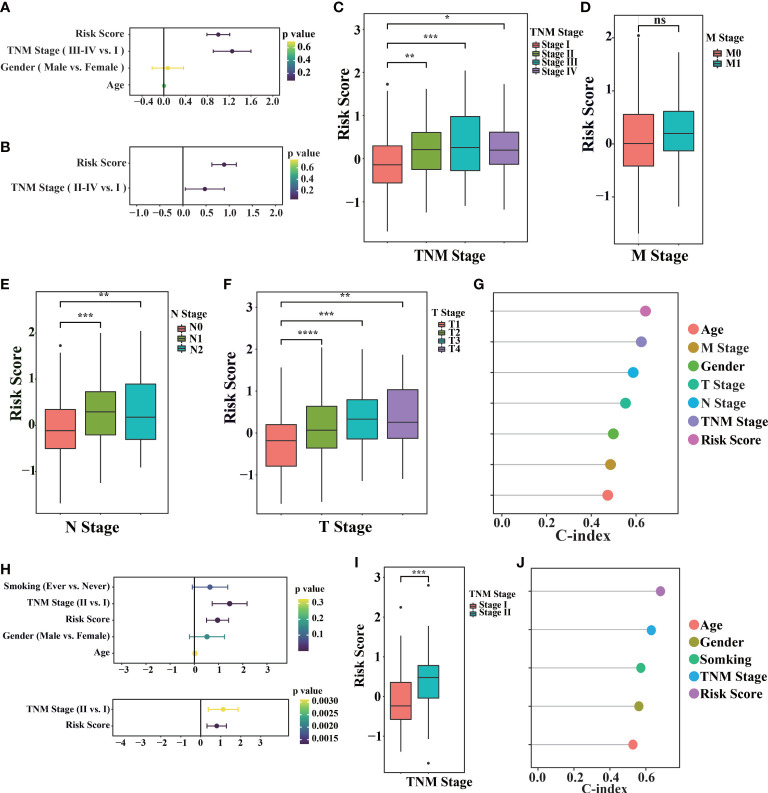
Prognostic values of the model in TCGA and GEO cohorts. **(A)** The TCGA-LUAD primary cancer samples, corresponding clinical records and corresponding risk score (n = 520) were used for univariate Cox regression. **(B)** Perform multivariable Cox regression on the significant factors in univariate Cox regression. **(C–F)** TCGA-LUAD samples with different tumor traditional clinical stage (TNM stage, M stage, N stage, T stage) and their corresponding risk scores. **(G)** Consistency index of TCGA-LUAD primary cancer samples, corresponding clinical records and corresponding risk score (n = 520) calculated by univariate Cox regression. **(H)** Using univariate Cox regression (above) in the GSE31210 cohort (n = 246), the significant factors in univariate Cox regression are performed in multivariable Cox regression (below). **(I)** samples with different TNM stages in GSE31210 cohort and their corresponding risk scores. **(J)** The consistency index of primary cancer samples, corresponding clinical records and corresponding risk scores in GSE31210 cohort was calculated by univariate Cox regression. (“ns” is non-significant, *P< 0.05, **P< 0.01, ***P< 0.001, ****P< 0.0001).

After controlling for confounding variables, risk score of the signature remained statistically significant for OS. The clinical characteristics analysis of the cohorts is summarized in **Table S1** and **Table S2**. As shown in **Figure 3A**, univariate Cox survival analysis indicated that risk score (p< 0.0001), invasion depth (T stage, p< 0.0001), distant metastasis (M stage, p< 0.05), lymph node metastasis (N stage, p< 0.0001) and clinical staging (TNM stage, p< 0.0001) were significant parameters that affect the prognosis of LUAD patients. Multivariable Cox survival analysis revealed that risk score was independent predictors of unfavorable prognosis in LUAD patients (p=4.33e-11 **Figure 3B**). Additionally, with LUAD TNM staging progress, the risk score increased in different degrees (**Figure 3C–F**). These results suggest that high-risk score might imply worse clinical symptoms regarding invasion depth, distant metastasis, and lymph node metastasis. By analyzing the risk score, we found that the model had a higher C-index, indicating that the risk score was more accurate than the traditional clinical stage (**Figure 3G**).

We also found the same result in GEO database (GSE31210, **Figures 3H–J**; GSE50081, **Figures S1G–K**), with LUAD TNM staging progress, the risk score increased in different degrees. These results indicate the risk scoring model, as an independent prognostic factor in clinical features, can more accurately evaluate prognosis of patients.

### Functional enrichment analyses of the prognostic signature

**Figure 4:**
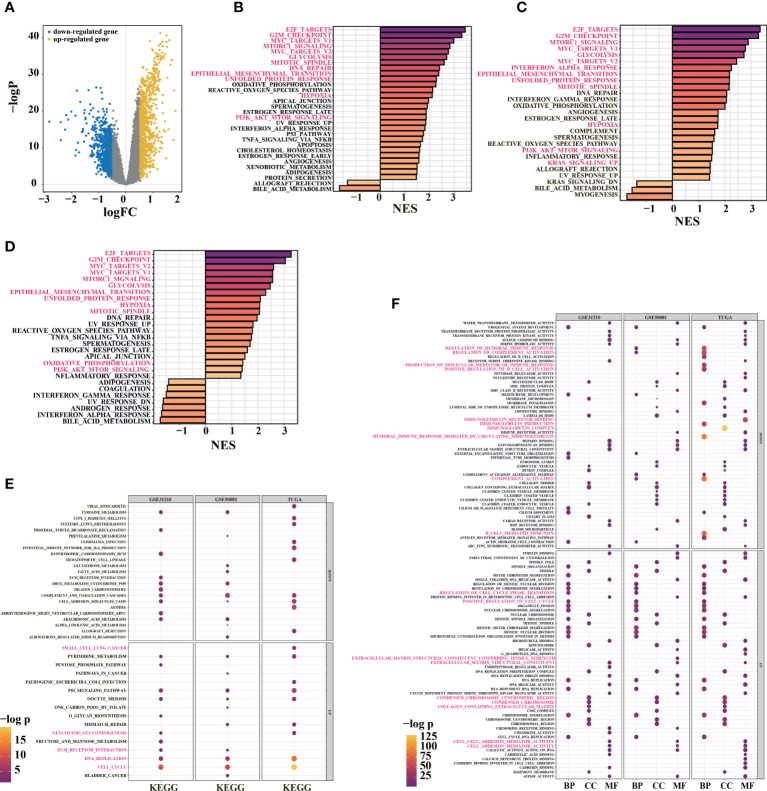
Functional enrichment analysis for prognosis signature. **(A)** volcano diagram show DEGs between high-risk and low-risk groups in TCGA-LUAD primary cancer samples. **(B)** GSEA enrichment analysis was performed using DEGs between high-risk and low-risk groups in TCGA cohort (30/50 genes were significant). **(C)** GSEA enrichment analysis was performed on the DEGs between high-risk and low-risk groups in the GSE31210 cohort (26/50 gene sets were significant). **(D)** 29/50 significant gene sets were obtained by GSEA enrichment analysis using DEGs between high-risk and low-risk groups in GSE50081 cohort (adj. pvalue< 0.05, color indicates the size of standardized enrichment score). **(E)** Based on LUAD samples in TCGA, GSE31210 and GSE50081 cohorts, the DEGs between high-risk and low-risk groups were analyzed for enrichment. Gene sets in KEGG database**(E)** and GO database**(F)** were used for enrichment analysis (FDR< 0.01; Color and dot size indicate enrichment significance).

To analyze the prognostic signature, base on differential analysis between high-risk and low-risk groups (**Figure 4A**), GSEA score on 50 hallmark pathways were displayed. Notably, we found some tumor-related pathways were activated in high-risk patients, such as epithelial-mesenchymal transition, mTORC1, and PI3K/Akt/mTOR pathways (TCGA, **Figure 4B**). The activation of mTORC1 signaling and PI3K/Akt/mTOR pathways promotes glucose metabolism and growth regulation (42). It is also worth noting that LUAD patients with epithelial-mesenchymal transition, glycolysis, and highly proliferative state were associated with poorer survival (43). The imbalance of these pathways may be related to tumor progression, indicating the poor prognosis in high-risk patients.

In TCGA and GEO cohorts (TCGA, **Figure 4B**; GSE31210, **Figure 4C**; GSE50081, **Figure 4D**), E2F transcription factors and c-Myc signal were activated in the high-risk group. The c-Myc signal regulates differentiation and proliferation through activated transcription and amplification of target genes in various tumors (44). In addition to mediating the cell cycle, E2F transcription factors play critical roles in tumor development and metastasis, including angiogenesis, extracellular matrix remodeling, tumor cell survival, and epithelial-mesenchymal transition (44, 45). With the activation of hypoxia-related pathways in high-risk group, unfolded protein responses are markedly activated (**Figures 4B–D**), and tumor cells may produce tumor-specific exosomes. Increased unfolded protein responses in high-risk group may contribute to ER stress. These factors jointly promote tumor proliferation, drug resistance, metastasis, and invasion and might even cause an immunosuppressive microenvironment (46).

Furthermore, “clusterProfiler” R package was used to conduct GO and KEGG enrichment analyses between high-risk and low-risk groups in TCGA and GEO cohorts (**Figures 4E, F**). Interestingly, KEGG pathway analysis indicated that the signature was associated with P53 signaling pathway, immune response, DNA replication, extracellular matrix remodeling (ECM)-receptor interaction, and glycolysis (**Figure 4E**). Similarly, the overlapped GO functional pathways between the three cohorts were predominantly enriched in tumor microenvironments associated with multivesicular bodies, mitotic activity, immune response, ECM, focal adhesion, and others (**Figure 4F**). These results imply that there might be differences in ER stress, exosome pathway, immune response, ECM, cell cycle, and proliferation between high-risk and low-risk groups.

### Analyze tumor microenvironment and validation differences biological process

**Figure 5:**
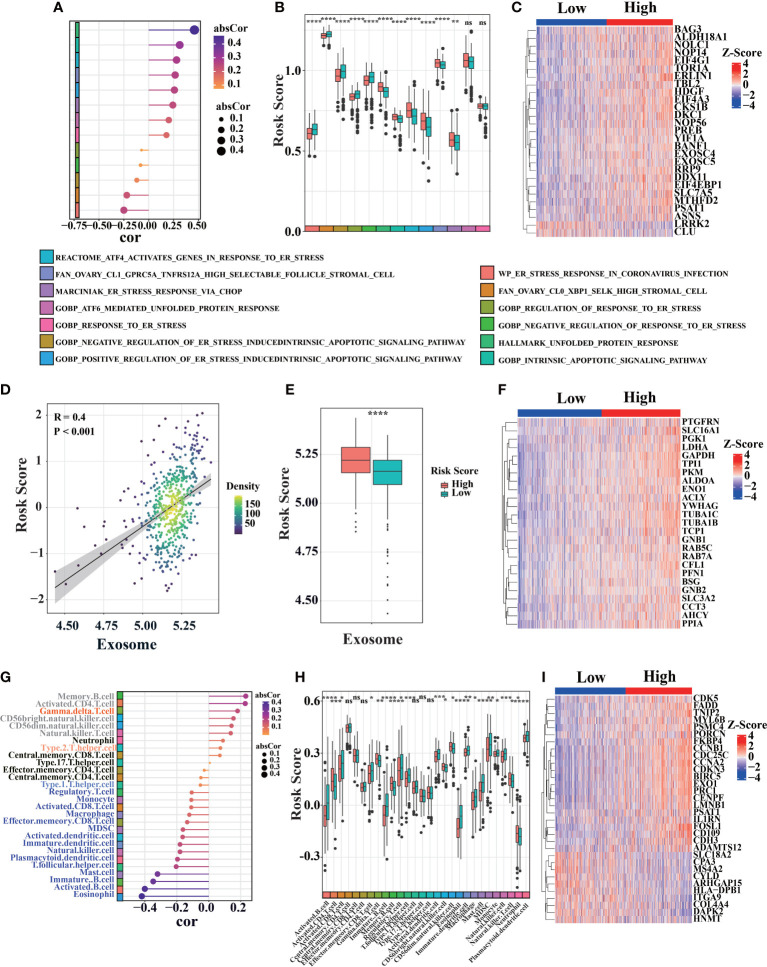
Bioprocess analysis base on prognosis signature. **(A)** Correlation diagram between risk score and ER stress-related gene set score (ssGSEA) in primary LUAD sample from TCGA cohort (n = 520). **(B)** Analyze ER stress-related gene set scores (ssGSEA) differences between high-risk and low-risk of primary LUAD samples in TCGA cohort. **(C)** Heat map of ER stress-related genes expression in LUAD patients from TCGA database. **(D–F)** The correlation between risk score and exosome associated score were analyzed similarly to the above ER stress pathway in TCGA cohort. **(G–I)** The correlation between risk score and different immune cell infiltration were analyzed similarly to the above ER stress pathway in TCGA cohort (the color block on the left represents different types of immune cells, the position indicates the magnitude of correlation coefficient, “ns” was non-significant, *P< 0.05, **P< 0.01, ***P< 0.001, ****P< 0.0001).

To further explore the differences in survival, we compared the effects of immune response, exosome pathway, and ER stress between two groups. It was observed that high-risk patients were associated with significantly higher ER stress scores (TCGA, **Figure 5A**; GEO, **Figure S2**). We also found that high-risk scores were related to some ER stress pathways (**Figure 5A**). Boxplots depicting ER stress-related ssGSEA scores showed that patients in high-risk group had higher scores than those in low-risk group (**Figure 5B**). To explore more detail about ER stress, a heatmap of ER stress-related genes was utilized to confirm the difference in both groups (**Figure 5C**). *Tbl2*, which is positively correlated with risk score, can cause ER stress through PERK-eIF2α-ATF4 axis (47) (**Figures 5C**, **S2B**). We also found that erlin1 and *EIF4EBP1* were positively correlated with risk scores (**Figure 5C**). ATF4-mediated induction of *erlin1* and EIF4EBP1 contributes to ER stress (48). As ER stress indicator, highly expressed *erlin1* indicates the increased ER stress in high-risk group (49) (**Figure S2C**). Therefore, ER stress is significantly involved in high-risk patients.

Meanwhile, we also found significant differences in exosome pathways. Compared with low-risk group, high-risk patients had higher exosome-related ssGSEA scores in TCGA (**Figure 5D**, additional data in GEO database, **Figure S3**). Cancer-derived exosome are implicated in various carcinogenesis processes, including malignant transformation, angiogenesis, immunosuppression, invasion, and treatment resistance (50, 51). There is also a significant correlation between exosome-related ssGSEA score and risk score in TCGA database (**Figure 5E**). Exosome-related genes also demonstrated different expression levels between two groups (**Figure 5F**). As a scaffold protein of exosomes, *PTGFRN* is positively correlated with risk score (**Figures 5F**, **S3B**) (52). In addition, we found that *ALDOA*, *ENO1*, *YWHAG* and *SLC3A2* were positively correlated with risk scores (**Figures 5F**, **S3C–F**). Previous studies have shown that *SLC3A2* has a higher level in lung cancer (53). Besides being the marker of exosomes, *ALDOA*, ***ENO1*** and *YWHAG* can promote metastasis, invasion, activation and proliferation of lung cancer (54–56).

In addition, low-risk patients were correlated with significantly higher immune scores in TCGA cohort (**Figure 5G**). Immune cells, as shown in **Figure 5H**, were found to be significantly difference between high-risk and low-risk patients in TCGA database. By immune signatures, we discovered that low-risk patients had higher immune infiltration than high-risk patients. Assessment of immune-related gene expression profiles, the characteristic immune-related genes such as FADD were selected based on their expression patterns between LUAD and the adjacent non-tumorous samples in TCGA database. Then, we compared the expressions of those selected immune-related genes between LUAD patients with high and low risk score, it suggested that immune infiltration plays a vital role in prognosis in LUAD patients (**Figure 5I**).

### The prognostic signature can identify the degree of malignancy

Moreover, we found that mutation frequencies of genes with cancer development, such as TP53, significantly differed between high-risk and low-risk groups (**Figure S5A**). Compared to TP53, where mutations are randomly distributed, the distribution of KRAS is relatively focused on KRAS-G12 locus mutations (called hotspot, **Figure S5B**). The combined distribution of TP53 and KRAS-G12 mutation showed that most significant difference between high-risk and low-risk groups (**Figure S5C**). In addition, 10 genes of the prognostic signature may have TP53 binding sites in their untranslated regions (UTR, upstream and downstream 1 kb of a gene), indicating that TP53 mutations might directly affect transcription of these genes (**Figure S5D**). Therefore, GEM with Kras-G12D with or without TP53 deletion were utilized to evaluate the LUAD prognostic signature.

**Figure 6:**
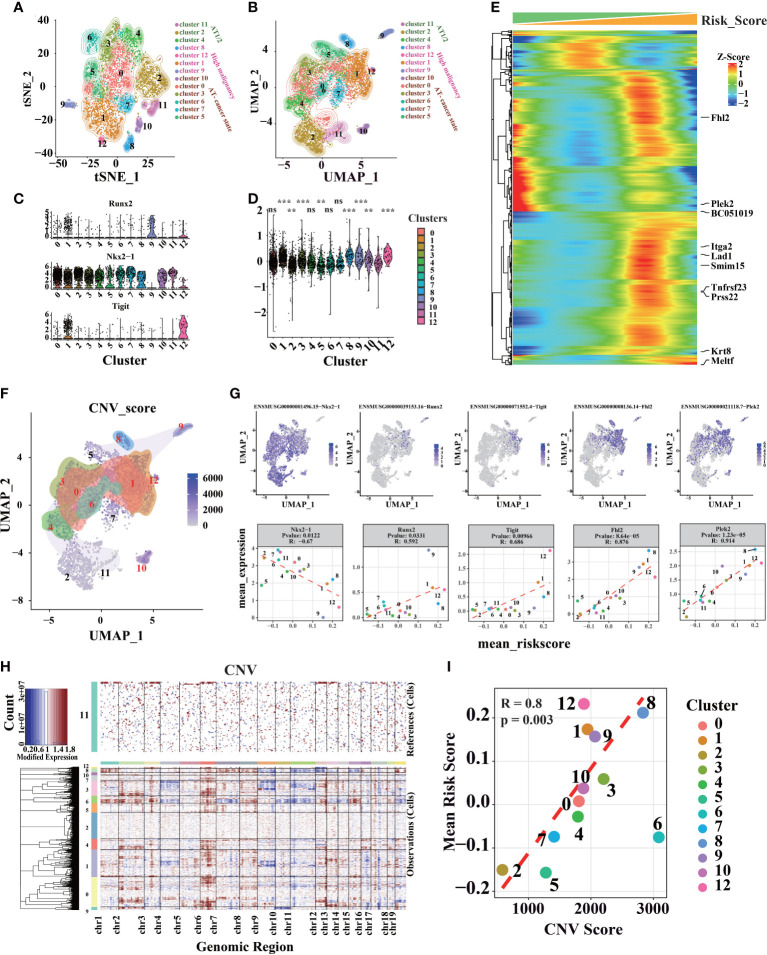
Different cell clusters identification and annotation in LUAD scRNA-seq data. **(A)** Seurat V4 was used to cluster and classify single cells, and t-SNE dimensional-reduction scatter diagram of sequencing data. **(B)** Sequencing data UMAP dimensional-reduction point scatter diagram. **(C)** Expression levels of genes associated with lung cancer development in different clusters. **(D)** The distribution of risk scores calculated by the prognostic signature in different cell clusters (“ns” is non-significant compared with cluster 11, *P< 0.05, **P< 0.01, ***P< 0.001, ****P< 0.0001). **(E)** genes associated with risk score (P.value< 10^-25^, the genes marked on the right are the 10 genes with the strongest correlation). **(F)** Distribution of CNV in UMAP dimensionality reduction map. **(G)** Genes associated with risk scores in scRNA-seq data. **(H)** InferCNV was used to predict the CNV of cells, and heat maps of the distribution of CNV of different chromatin of different cells. **(I)** Average CNV score and risk score points from different clusters are scattered figure.

Three thousand eight hundred ninety-one high-quality, full-length single-cell transcriptomes from 39 KrasG12D/+Trp53- mutation mice, at 8 distinct LUAD evolution stages starting with normal alveolar type 2 cells (AT2) and ending with fully formed LUAD, were downloaded from GSE154989. Additionally, Seurat V4 was used for clustering cells (57) (**Figures 6A, B**). Stemness-related genes (*Tight*, *Runx2*, and *NKX2-1*) revealed that cells in cluster 1, 9, and 12 have high stemness (**Figures 6C**, **S6A–C**). After calculate risk score of different clusters (**Figure 6D**), we found cluster 1, 9, and 12 had higher risk scores, which was consistent with stemness-related genes in different clusters. Furthermore, the correlation between risk score and tumor progression is positive (**Figures S6C–F**).

However, we found that cluster 3, 8, and 10 also had higher risk scores than others (**Figures 6D**, **S6G–J**). Therefore, feature genes were identified to evaluate the model. Cluster 3 has a higher *Vcan* and *Dcpp1*, which involve cell adhesion, proliferation, migration, and angiogenesis, and plays a central role in tissue morphogenesis and maintenance (58, 59). Cluster 10 has a higher level of *Ccnb1* and *Top2a*, which related to cell cycle and cell proliferation (60, 61). Cluster 8 has a higher level of *Rn7sk*, which was associated with Gastric Cancer (62) (**Figure S6A**). *Fhl2*, *Plek2*, and other cancer-related genes have a higher correlation coefficient with risk score (63–65) (**Figure 6E**). CNV was estimated using scRNA-seq data (**Figure 6F**) in contrast to cluster 11, as cluster 11 only appeared in the early stage of tumor progression (**Figures S6B, C**). Each cluster’s average risk score is associated with the average CNV score; for instance, malignant cells usually have serious CNV (**Figures 6G–I**). On the other hand, we found that CNV was higher in KP mice after 12 weeks (**Figures S6K–M**). It shows that the intracellular CNV accumulates with tumor progression, and tumor with higher CNV indicates a higher degree of malignancy, consistent with these results described through the risk score. Therefore, cells with higher risk scores have higher CNV and a higher degree of malignancy.

### Validated the biological process of prognostic model by scRNA-seq

**Figure 7:**
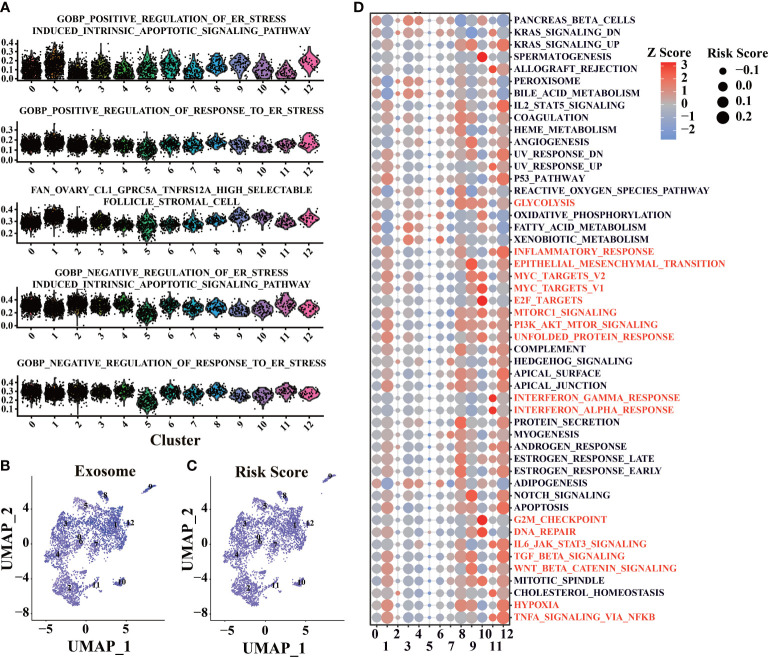
Functional enrichment of scRNA-seq base on the prognosis signature. **(A)** Enrichment analysis of ER stress-related gene sets was conducted by AUCell software. **(B)** Enrichment analysis of exosome related gene sets was conducted by AUCell software. **(C)** Use the prognostic signature to calculate the risk score of cells. **(D)** Enrichment analysis of tumor-associated Helmark gene sets was performed using AUCell.

To proving the details of risk score in tumor microenvironment, AUCell calculated the score in ER stress and exosome-related gene sets by scRNA-seq data. As expected, ER stress scores (**Figure 7A**) and exosome scores (**Figure 7B**) were higher in cluster 1, 9, and 12, which have higher risk scores. There is also a high consistency between exosome score and risk score (**Figure 7C**), correlating with our earlier findings.

Functional enrichment was performed in different clusters with hallmark gene sets to evaluate each cell’s biological process (**Figure 7D**). Cluster 10 has a higher activation of E2F targets and G2M checkpoint, leading to higher risk scores. Cluster 1, 8, 9, 10, and 12 have a higher level of PI3K-AKT-mTOR, which could lead to increased risk scores. Interestingly, cluster 11, which only appeared in the early stage of tumor progression, had higher risk scores than cluster 2, 5, and 6. It suggests that immune response in the early stage of LUAD could promote tumor progression (66) (**Figure 7D**). Cluster 8 and cluster 12, which have high-risk scores and mostly appeared in the middle stages of LUAD (12w and 18w, **Figures S6C–F**), showed more cellular response (such as IL2, IL6), and the abilities were lost with tumor progression, which could be related to the tumor progression (**Figure 7D**).

### Tumor progression can be described by risk score

To explore the relationship between risk score and tumor progression, LUAD marker genes and lung epithelial marker genes were calculated (as “bioscore”) through UMAP to visualize tumor progression and compared with risk scores (**Figure S7A**). We found that LUAD-related markers were concentrated in high-risk regions, while more lung epithelial marker genes were concentrated in low-risk regions. The scRNA-seq data of different genotypes and experimental time were calculated using dimensionality reduction by UMAP. It was found that high-risk cells were concentrated in the region of KRAS and Trp53 mutations and mainly distributed in the late stage of the experiment. Meanwhile, cluster 1, 8, 9, and 12 were primarily distributed in the high-risk region. According to the pseudotime of Monocle V3, the region is also the accumulation of advanced LUAD cells (**Figure S7B**).

Furthermore, we found that risk score was positively correlated with most LUAD marker genes and negatively correlated with lung epithelial marker genes (**Figure S7C**). These results indicate that risk score can describe the development process of lung epithelial to LUAD cells.

**Figure 8:**
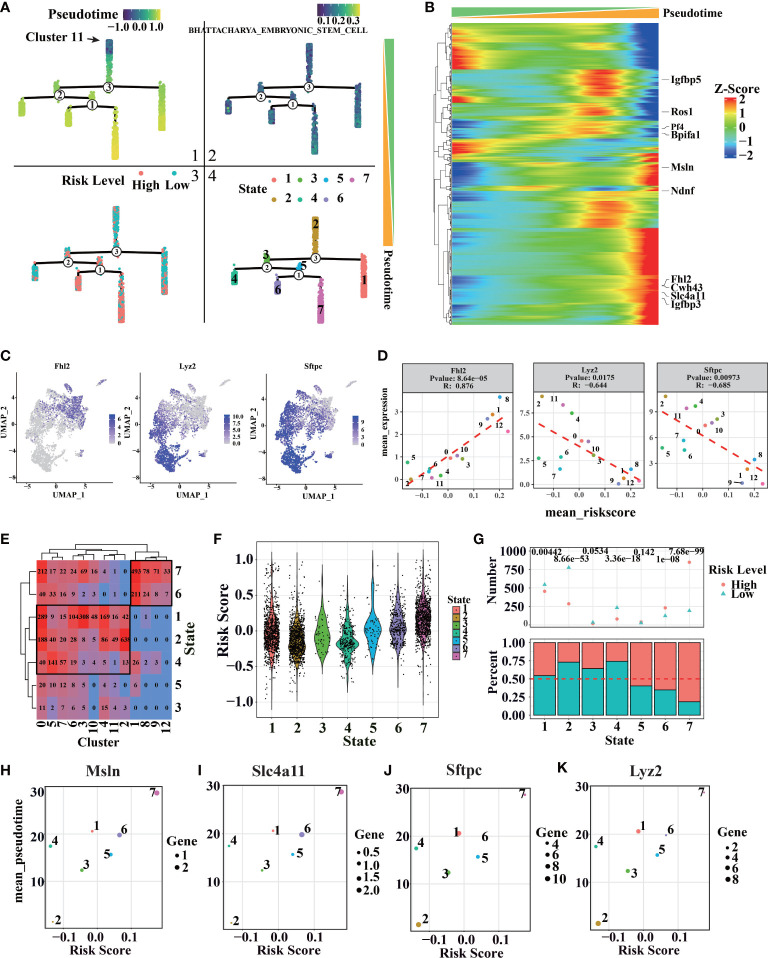
Cell trajectory and pseudotime analysis of LUAD by scRNA-seq. (A1) Distribution of pseudo-time values of different cells in the minimum spanning tree. (A2) The distribution of BHATTACHARYA_EMBRYONIC_STEM_CELL score in the minimum spanning tree. (A3) Distribution of risk rating in the minimum spanning tree. (A4) Distribution of different cell states in the minimum spanning tree. **(B)** Genes related to pseudo-time series, the genes marked on the right are the 10 genes with the strongest correlation. **(C, D)** Distribution and correlation of pseudo-time related genes and branching related genes. **(E)** The relationship between cell states and clusters in different pseudo-time series, the number represents the number of cells in the corresponding state. **(F)** Cell risk values corresponding to different quasi-sequential states. **(G)** Ratio of cell risk states corresponding to different pseudo temporal states (above: the top number represents the significance of binomial distribution test; below: the ratio of high-risk cells to low risk cells in each state). **(H)** Scatter plot of *Msln* expression mean and risk mean of cells in different states. **(I)** Scatter plot of *Slc4a11* expression and risk score of cells in different states. **(J)** Scatter plot of *Sftpc* expression and risk score of cells in different states. **(K)** Scatter plot of *Lyz2* expression average and risk average of cells in different states.

For more details of tumor progression, high variable genes characterized by monocle V2 were used for estimating pseudotime. Cluster 11, which appeared in the early stage of LUAD, was set to the initial point of pseudotime (**Figure 8A1**). The minimum spanning tree, “Stemness” related genes downloaded from ‘Msigdb’, can also estimate pseudotime correctly (**Figure 8A2**). With tumor progression, malignant cells with strong stemness appear in LUAD, which have the potential for multi-directional differentiation, leading to intratumoral heterogeneity (67, 68). Therefore, LUAD progression should be considered in stemness levels by “Stemness” related genes (**Figures S9, S10B**). *Igfbp5*, *Ros1* (54–56), and other genes related to tumor progression profoundly correlate with pseudotime (**Figure 8B–D**), meaning the minimum spanning tree and pseudotime can describe tumor progression in more detail. With the increase of pseudotime, the malignant degree of tumor and the proportion of high-risk cells also increased, indicating that the risk score model can reflect more advanced tumor progression (**Figure 8A3**). Notably, seven states have been classified by a minimum spanning tree (**Figure 8A4**). Previously, we found that cluster 1, cluster 8, cluster 9, and cluster 12 have higher risk scores. According to the clusters, states 6 and 7, located under branch 1, also have high-risk scores (**Figures 8E–G** and **S8A**).

More pseudotime-related genes and branch-related genes were recognized (**Figures 8B**, **S8C–E**). Pseudotime related genes *Msln* and *Slc4a11* can promote tumor progression (69, 70). Additionally, branch-related genes *Sftpc* and *Lyz2*, known as AT2 markers (19, 57), are shown as typical examples (**Figures 8H–K**, **S10A**). The genes can reflect tumor malignant degree and further describe the similarity and branching relationship in tumor clusters. So, we can infer that the AT2 cell features were missing with the cancer progress, accompanied by increased LUAD features (**Figure S7**). As we expect, the mean pseudotime and risk score of different states have a severe positive correlation. Interestingly, the mean expression of pseudotime-related genes and branch-related genes is used to clearly different states more clearly, showing some key points during tumor progression. Furthermore, a risk score can help us estimate the malignant of each state.

## Discussion

LUAD is a complex disease in which multiple pathways are involved in pathogenesis. Exploring a novel accurately prognostic biomarkers would help select patients for adjuvant chemotherapy and improve prognosis in early-stage lung cancer. We build a prognostic model in TCGA cohort by LASSO regression, which has excellent specificity and sensitivity for predicting OS. To identify whether risk score as an independent predictor of survival time, univariate and multivariable cox proportional hazards regression was analyzed in LUAD patients. After controlling for confounding variables (including age, gender, invasion depth, distant metastasis, lymph node metastasis, and TNM stage), the model remained statistically significant for OS. The model can be an independent factor with better predictive potential than the pathological stage alone.

Several factors have been proved to be related to tumor progression; however, only hypoxia and glycolysis were highly associated with LUAD prognosis (71, 72). Here, we found risk score was related to several biological processes like epithelial-mesenchymal transition, ECM, glycolysis, and proliferation, which were involved in tumor progression. The prognostic model could reveal the critical elements involved in tumor microenvironments. More details about ER stress, exosome, and immune response, which play essential roles in tumor progression, were researched, and we found that risk score relates to these biological phenomena. The prognostic differences between high and low-risk groups could be explained by the critical elements involved in tumor microenvironments (71, 72). Recently, cancer-released exosomes could modify the distant microenvironment to a pre-metastatic niche to facilitate the formation of metastatic lesions, suggesting that cancer exosomes could result in both local and distant effects (73, 74), including malignant transformation, angiogenesis, immunosuppression, invasion, and treatment resistance (51, 75). The unfavorable intratumoral microenvironment, such as nutrient deficiency, hypoxia, high metabolic demand, oxidative stress, and unfolded protein response, can also ultimately induce ER stress, enhancing tumorigenicity, metastasis, and tumor drug resistance. It is reported that ER stress regulates proliferation, migration, and invasion through active c-Myc signaling and PI3K/AKT/mTOR signaling pathways, and mediated anti-tumor immune responses by inducing immunosuppressive microenvironment (76–78). We found that low-risk patients correlated with a significantly higher immune score in TCGA cohort, and immune cell populations are different between high-risk and low-risk patients in TCGA database. Therefore, risk score could be used to evaluate immune infiltration in LUAD patients, which may be useful for immune-targeted tumor therapy. It is well known that tumor microenvironment can be classified into two immunophenotypes based on their degree of immune infiltrations, hot tumors with high immune infiltration and cold tumors with low immune cell infiltration (79, 80). Low-risk patients with a high immune score suggest the presence of a hot tumor microenviroment, those patients could benefit more from immune-targeted therapy than high-risk patients with cold tumor microenviroment. However, further studies are still needed to confirm the prognostic value of risk score in determining hot/cold tumors.

By previous studies, we found some prognostic signatures may have poor repeatability due to insufficient sample size (81, 82). One limitation of this study is the relatively small sample and the poor quality of the GEO cohort, which was used to verify the prognostic signature. To address this issue, we have used multiple GEO cohorts to further verify the prediction performance of the prognostic signature. Further, tumor progression can also be influenced by complex and dynamic features in tumor surroundings, which means a model based on several gene sets may lead to bias. To develop a good prediction model for OS, GEM were used to test the model in different dimension through scRNA-seq data. By single-cell clustering, stemness-related genes (*Tight*, *Runx2*, and *NKX2-1*) revealed that cells in cluster 1, 9, and 12 have high stemness. Tight, a marker of high-plasticity cell, shows high proliferative potential and can be induced chemoresistance (57). *Runx2* can drive the metastatic phenotype in the primary tumors, and *NKX2-1* also shows the same consequence (83–85). Furthermore, the correlation between risk score and tumor progression is positive. According to tumor-related feature genes, we found that clusters 3, 8, and 10 also have higher risk scores than other clusters. Cluster 3 was involved in cell adhesion, proliferation, migration, and angiogenesis and played a central role in tissue morphogenesis and maintenance (58, 59). Cluster 10 has a higher level of cell cycle and cell proliferation (60, 61), and cluster 8 was related to Gastric Cancer (62). In conclusion, we found that the higher risk score, the higher degree of malignancy. In addition, risk score in each cluster is associated with average CNV score, such as malignant clusters usually have serious CNV. However, cluster 6 has a lower risk score and higher CNV level, which might be induced by intratumoral heterogeneity.

Furthermore, we found that risk score was related to tumor progression calculated by Monocle. Minimum spanning tree has been applied to describe LUAD progression, which was confirmed by enrichment analysis on ‘BHATTACHARYA EMBRYONIC STEM CELL’ collected from Msigdb (86). With the advancement of LUAD, risk score and the proportion of high-risk cells were increasing. We also notice that cells under branch2 (cells in stat6 and stat7) show higher risk scores. Moreover, we found several AT2 marker genes (*Sftpc* and *Lyz2*) at the branch2, which means that branch2 is a crucial point for LUAD progression. As we expect, the pseudotime and risk score of different states have a severe positive correlation. Interestingly, pseudotime-related genes and branch-related genes is used to clearly different states more clearly, showing some key points during tumor progression. Overall, the risk score was in accordance with the grade malignancy in each cluster, which was annotated by feature genes, pathways related to tumor progress, CNVs and genotype, and growth time of GEM. Based on this, our follow-up research will focus on clinical application and molecular mechanisms.

In conclusion, the study established a risk scoring model, which can be used as an independent prognostic signature to accurately evaluate the prognosis of LUAD patients. Compared with traditional clinical indicators, the model has higher accuracy and stability, and can provide guidance for follow-up treatment. The prognostic signature related to several biological processes, which may reveal the key molecular mechanisms in tumor development.

## Data availability statement

The datasets presented in this study can be found in online repositories. The names of the repository/repositories and accession number(s) can be found in the article/**Supplementary Material**.

## Author contributions

ZL, TZ and WY conceived and designed the study. ZL drafted the entire manuscript. TZ is responsible for LUAD patient data acquisition and bioinformatics support. ZL and CZ participated in figures and table preparation. WY and YC reviewed and revised the manuscript. TZ and CZ guided the selection of statistical methods. WY and YC supervised the project. All authors contributed to the article and approved the submitted version.

## Funding

This project was sponsored by the Natural Science Fund of China (81673462, 82073916, 81874452, and 91540119) and the Key development project of Jiangsu Province (BE2017712).

## Acknowledgments

The authors thank AiMi Academic Services (www.aimieditor.com) for the English language editing and review services. In addition, ZL would like to thank Lan Zhang for the ongoing and unwavering support, patience, and love.

## Conflict of interest

The authors declare that the research was conducted in the absence of any commercial or financial relationships that could be construed as a potential conflict of interest.

## Publisher’s note

All claims expressed in this article are solely those of the authors and do not necessarily represent those of their affiliated organizations, or those of the publisher, the editors and the reviewers. Any product that may be evaluated in this article, or claim that may be made by its manufacturer, is not guaranteed or endorsed by the publisher.
