# Supplementary material for: A prognostic signature model for unveiling tumor progression in lung adenocarcinoma
Source: Front Oncol. 2022 Nov 1;12:1019442. doi: 10.3389/fonc.2022.1019442 (PMC9663930; doi:10.3389/fonc.2022.1019442)
Supplement: Supplementary file 1 [file DataSheet_1.docx]

Supplementary Material

# Supplementary Figures and Tables

## Supplementary Figures

**
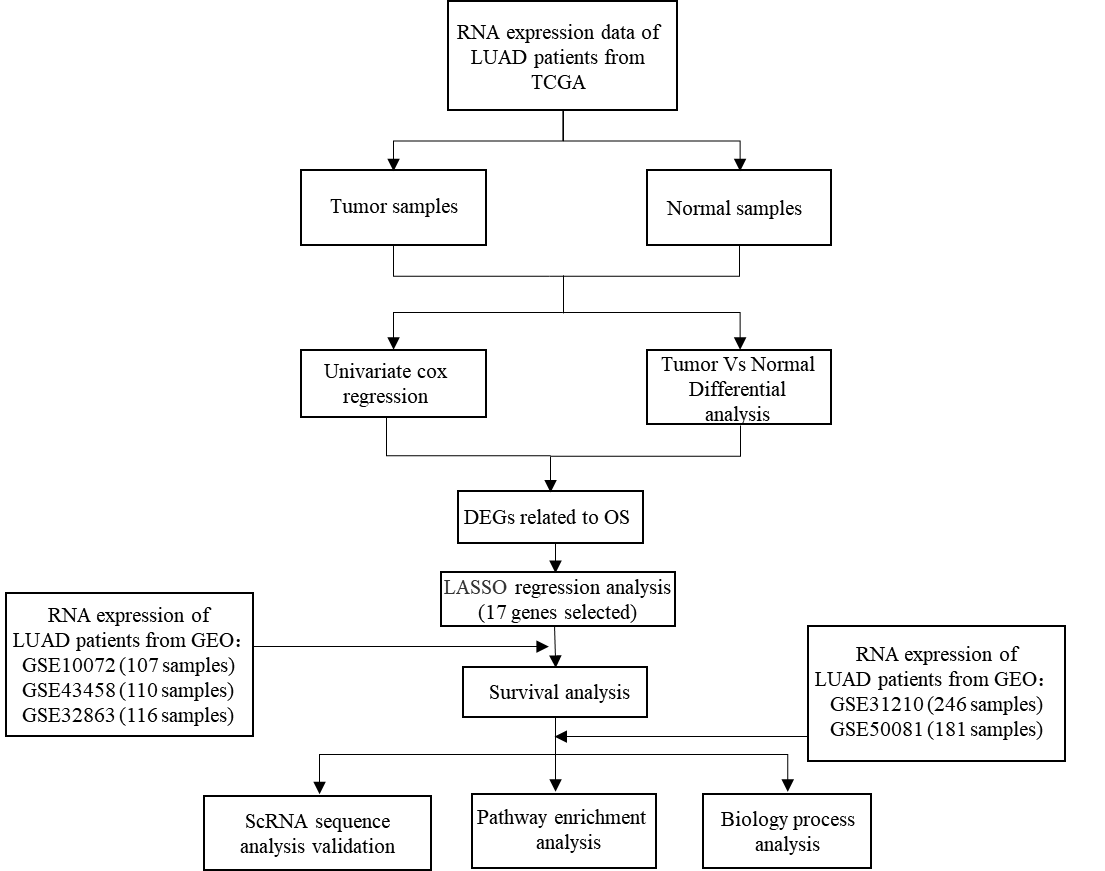
**

**Flow chart: the general pipeline of the data analysis protocol.**


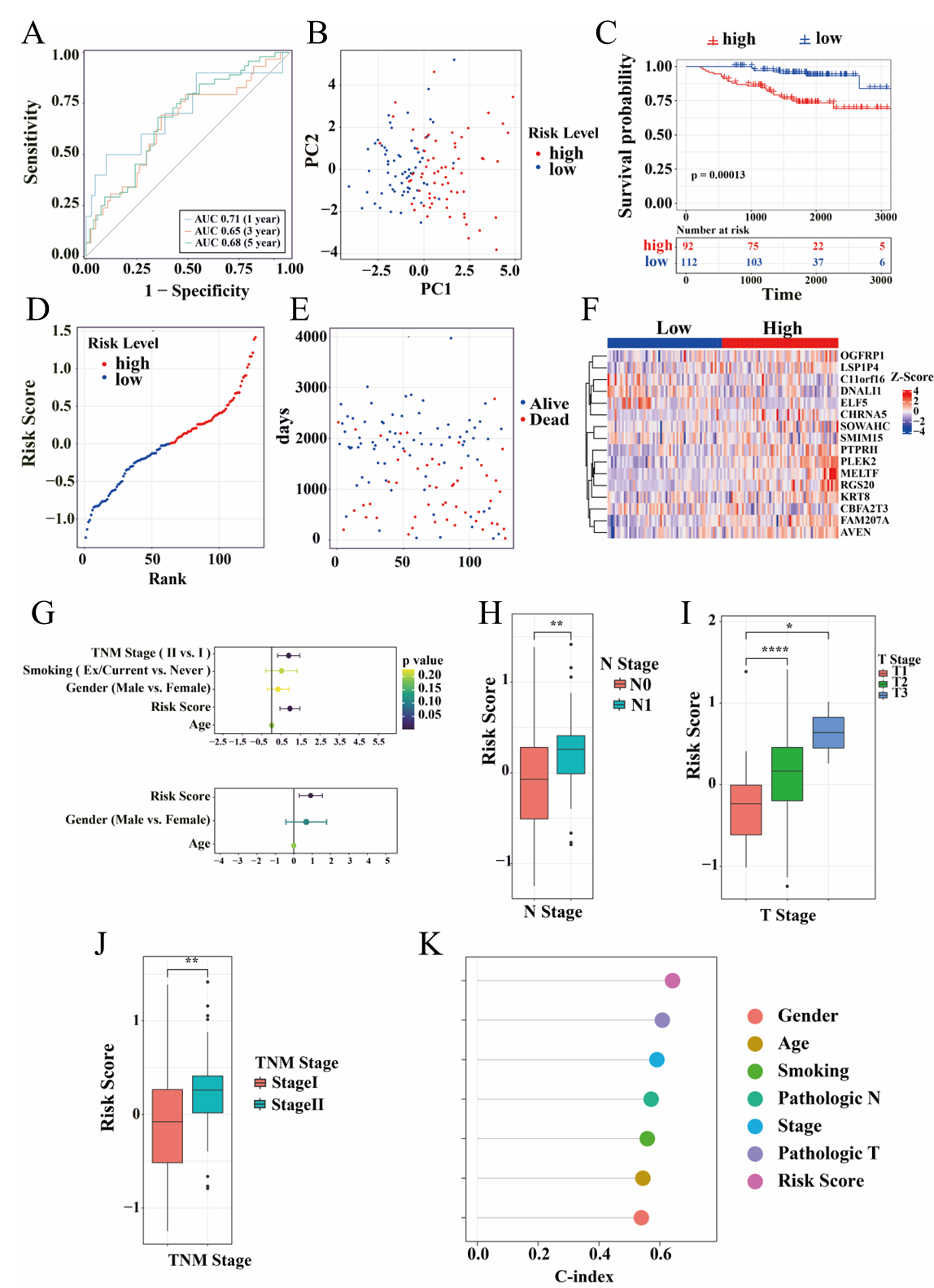


**Supplementary Figure 1.** Survival analysis of the model in GEO database (GSE50081 cohort). (A) The ROC curve of risk score calculated from cancer samples and corresponding clinical records in GSE50081 cohort (n = 181) at 1, 3 and 5 years. (B) PCA analysis was performed on the genes obtained in Figure 2.1c of cancer samples in GSE50081 cohort.  (C) KM survival analysis was performed to test the difference in survival rates of patients with high and low risk groups in GSE50081 cohort. (D) Distribution map of different survival risks in GSE50081 cohort. (E) Distribution of time to death of patients with different survival risks in GSE50081 cohort (ordinate: follow-up time, abscissa: risk ranking). (F) Gene expression trend in Figure 1C in GSE50081 cohort (top note: blue: low risk, red: high risk). (G) Univariate Cox regression was performed using cancer samples and corresponding clinical records and risk scores in GSE50081 cohort (n = 181, above).  Multivariate Cox regression was performed for the significant factors in univariate Cox regression (under, upper and lower limits were 95% confidence intervals, and color was significance). (H) samples with different primary stages (TNM stage) in GSE50081 cohort and their corresponding risk scores. (H) Risk scores of different lymph node staging samples in GSE50081 cohort (different colors indicate different lymph node staging). (I) Samples with different tumor stages in GSE50081 cohort and their corresponding risk scores. (J) samples with different primary stages in GSE50081 cohort and their corresponding risk scores. (K) The consistency index was calculated by univariate Cox regression for tumor samples and corresponding clinical records and risk scores in the GSE50081 cohort (“ns” was non-significant, * P < 0.05, * * P < 0.01, * * * P < 0.001, * * * * P < 0.0001).


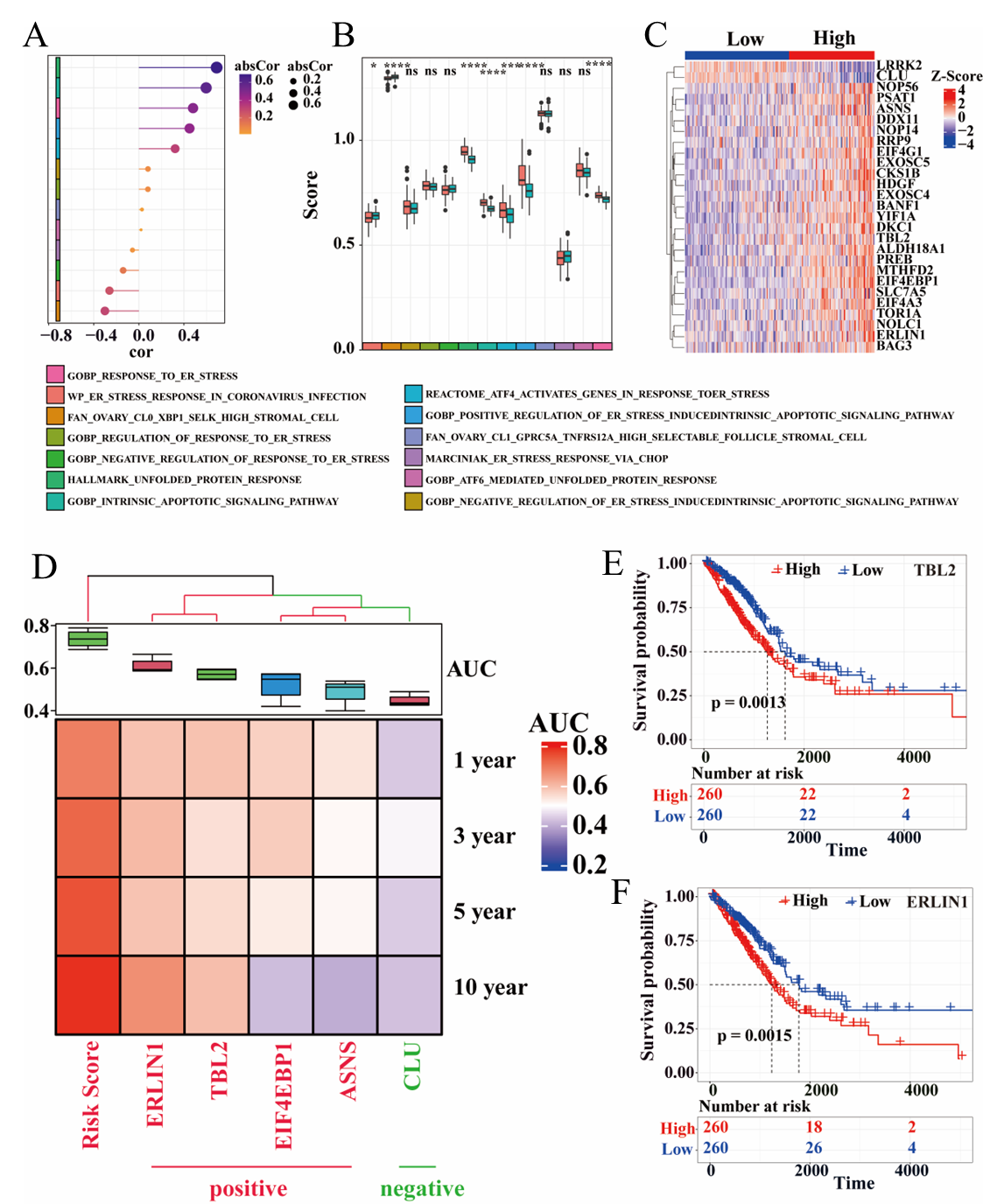


**Supplementary Figure 2.** Endoplasmic reticulum stress analysis in GEO database. (A) Correlation diagram between primary cancer sample risk score and ER stress-related gene set score (ssGSEA) in GSE31210 cohort (n = 246, the color on the left represents different ER stress-related gene sets, and the color and size of the dot represent the correlation coefficient). (B) Analysis of differences in ER stress-related gene set scores (ssGSEA) between high and low-risk of primary cancer samples in GSE31210 cohort (bottom note: different colors indicate different ER stress-related gene sets). (C) Heat map of er stress-related gene expression in LUAD patients from GSE31210 cohort (“ns” was no-significant, * P < 0.05, * * P < 0.01, * * * P < 0.001, * * * * P < 0.0001). (D) ER stress-related genes were used to predict the prognosis at different time points, and the AUC value obtained was time-dependent on ROC curve. (E-F) The samples were divided into high expression and low expression according to the median of the genes (TBL2, ERLIN1) expression level, and KM analysis was performed (red: the gene expression level was higher than the median in those samples, blue: the gene expression level was lower than the median in the samples).


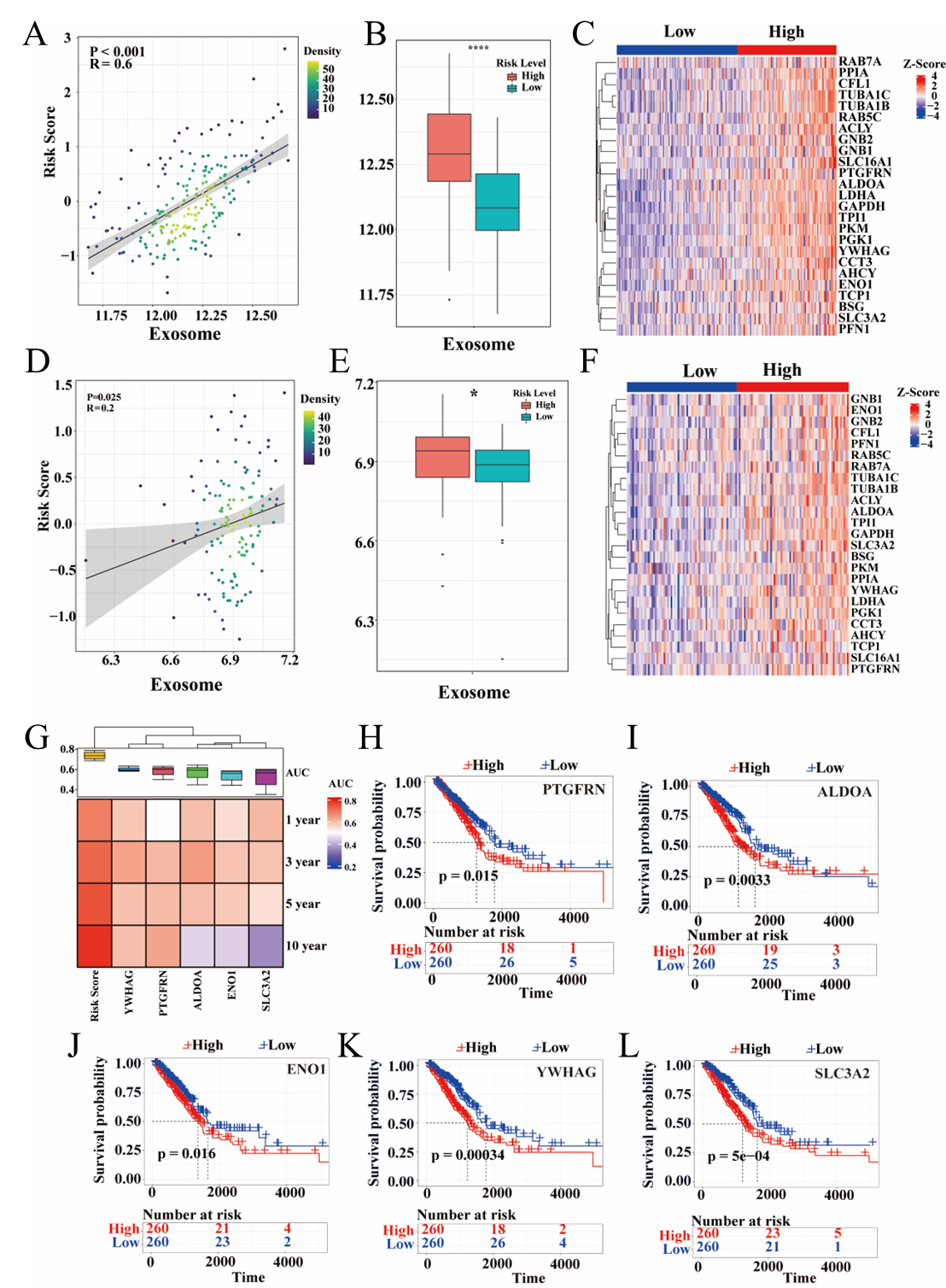


**Supplementary Figure 3.** Exosomes analysis in GEO database. (A) Correlation diagram between primary cancer sample risk score and exosome associated gene set score (ssGSEA) in GSE31210 cohort (n = 246). (B) Analysis of difference in exosome associated gene set score (ssGSEA) between high and low risk of primary cancer samples in GSE31210 cohort. (C) Heat map of exosome related gene expression in LUAD patients from GSE31210 cohort (top note: blue: low risk, red: high risk). (D) correlation diagram between primary cancer sample risk score and exosome-associated gene set score (ssGSEA) in GSE50081 cohort (n = 181). (E) Analysis of difference in exosome associated gene set score (ssGSEA) between high and low risk of primary cancer samples in GSE50081 cohort. (F) Heat map of exosome related gene expression in LUAD patients from GSE50081 cohort (top note: blue: low risk, red: high risk). (G) The prognosis at different time points was predicted by exosome genes, and the obtained time depended on the AUC value of ROC curve. (H-L) The samples were divided into high expression and low expression according to the median of the genes (PTGFRN, ALDOA, ENO1, YWHAG, SLC3A2) expression level, and KM analysis was performed.


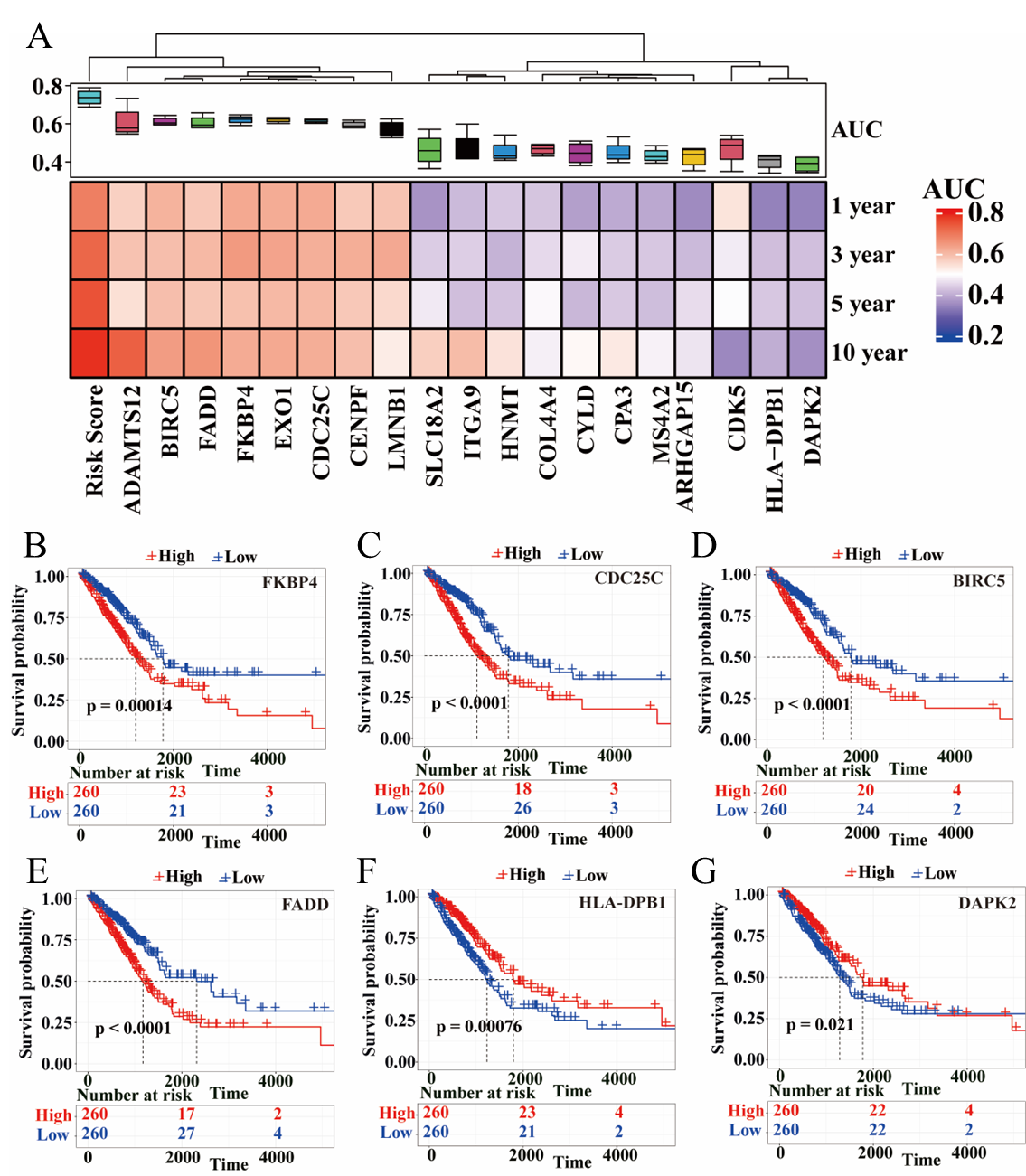


**Supplementary Figure 4.** Tumor immune microenvironment analysis of GEO database. (A)Using immune-related genes to predict prognosis at different time points, the AUC value of the time-dependent ROC curve obtained. (B-G) The samples were divided into high and low expression according to the median expression (FKBP4, CDC25C, BIRC5, FADD, hLA-DPB1, and DAPK2), and KM analysis was performed (red: genes expression level was higher than the median level, blue: gene expression level was lower than the median level).


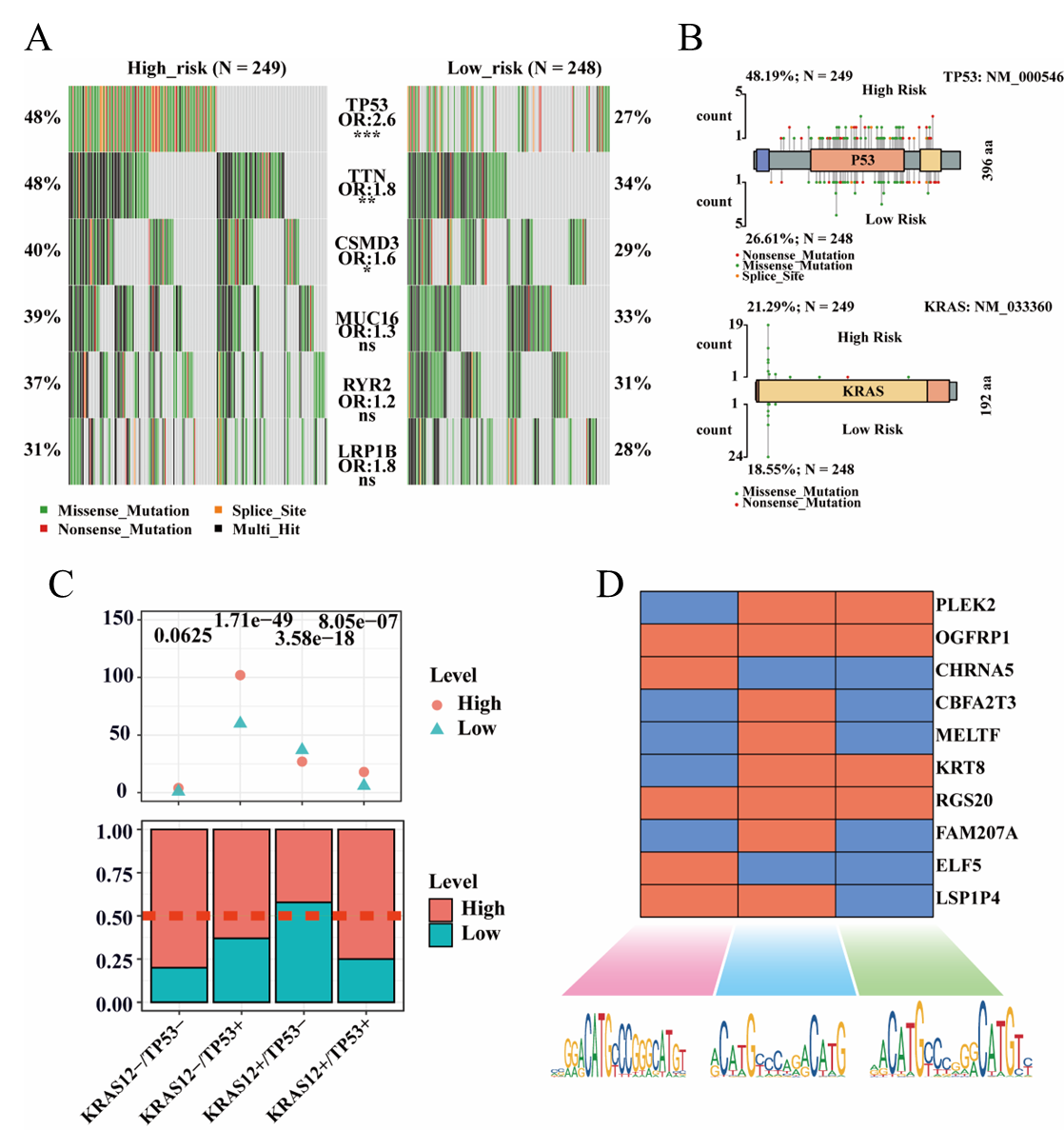


**Supplementary Figure 5.** The mutation distribution of TCGA database. (A) “Maftools” was used to calculate TCGA mutation data to obtain genes with High mutation frequency (High risk on the left: patients with High risk in TCGA; Low risk on the right: patients with Low risk in TCGA). (B) Mutation distribution of high-risk and low-risk patients with TP53 (above) and KRAS (below) in TCGA database (the above part represents high-risk mutation distribution and the below part represents low-risk distribution). (C) The combined distribution of KRAS-G12 and TP53 mutations (above: the top number represents the significance of the binomial distribution test, red circle: high risk, green triangle: low risk; below: the ratio of high risk cells to low risk cells in each state, red: high risk, green: low risk, and the red dotted line indicates the value of 0.5). (D) P53 binding motifs in the TSS region (above and below 1kbp) of the genes associated with survival risk (obtained in Figure 1C) (red: presence of binding motifs, blue: absence of binding motifs). (NS was not significant, *P < 0.05, **P < 0.01, ***P < 0.001) P53 binding motif in transcription start site region (upper and lower 1kbp) of genes associated with survival risk (red: presence of binding motif, blue: no binding motif).


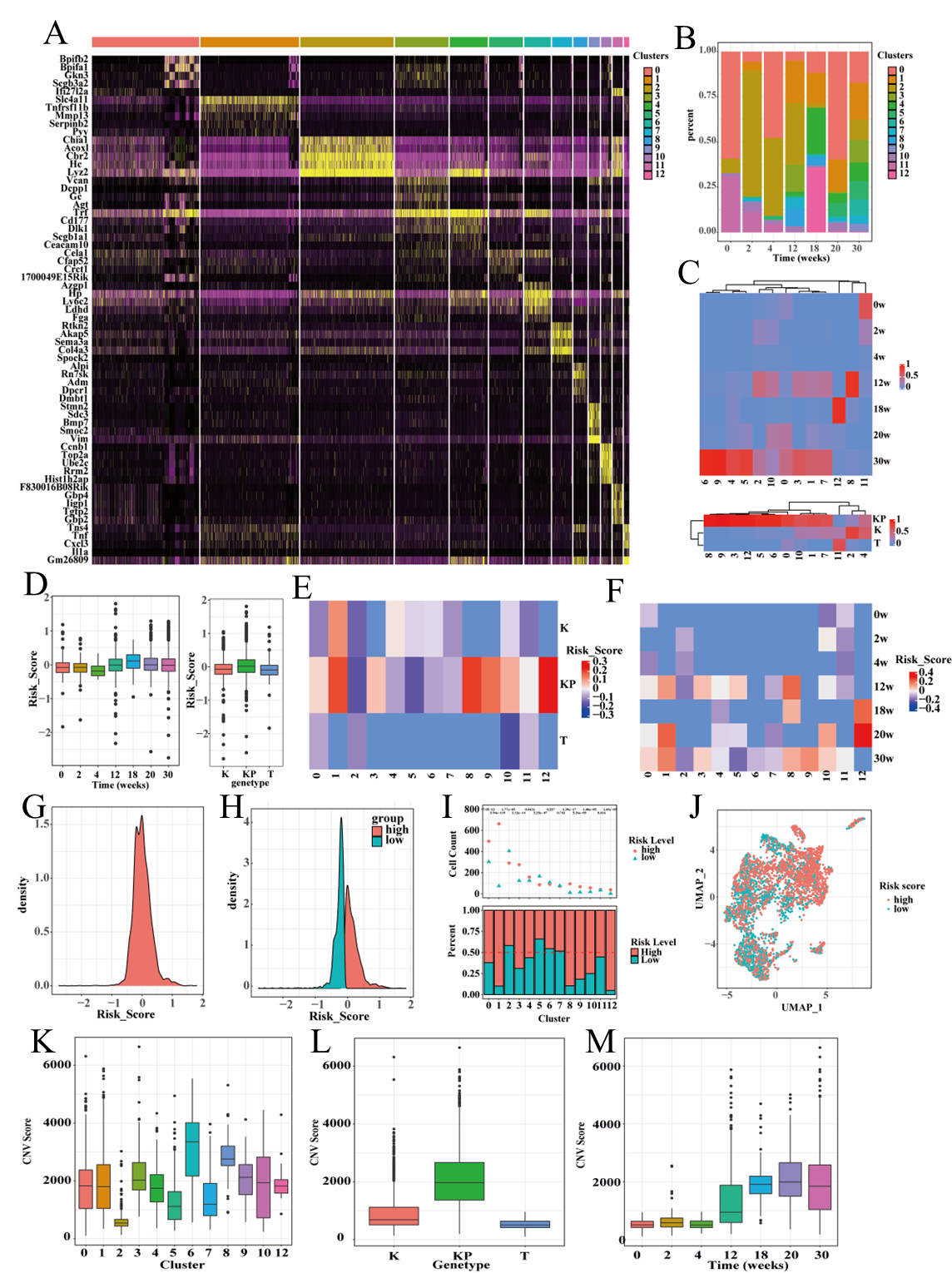


**Supplementary Figure 6.** Overview of scRNA-seq data. (A) Seurat V4 was used to cluster single cells and characterize gene expression in different clusters. (B) Cell composition at different experimental times (different colors indicate different clusters). (C) Distribution of different clusters. Above: the distribution at different time points (color indicates the relative number of clusters at different time points). Below: the distribution among different genotypes (color indicates the relative number of clusters among different genotypes, K: KRAS-G12D mutation, KP: KRAS-G12D and Trp53 double mutation, T: control group). (D) The analysis of risk score. Left: at different experimental times. Right: among different genotypes. (E) The effect of different clusters and genotypes on average risk score (red: high risk score, blue: low risk score, cornweed blue: cell percentage less than 0.026). (F) The effect of different clusters and different experiment time on average risk score (red: high risk score, blue: low risk score, cornflower blue: cell percentage less than 0.026). (G) Distribution of all cell risk scores. (H) Divide the cells into high risk and low risk according to their risk score. (Red: high risk, risk score greater than -0.1, green: low risk, risk score less than -0.1). (I) Number of high risk cells and low risk cells in different clusters (red: high risk, risk score > -0.1, green: low risk, risk score < -0.1). (J) Distribution of high-risk and low-risk cells (red: high risk, risk score greater than -0.1, green: low risk, risk score less than -0.1). (K) Distribution of copy number variation score in different clusters.  (L) Distribution of copy number variation in different genotypes.  (M) Copy number variation distribution at different experimental times.


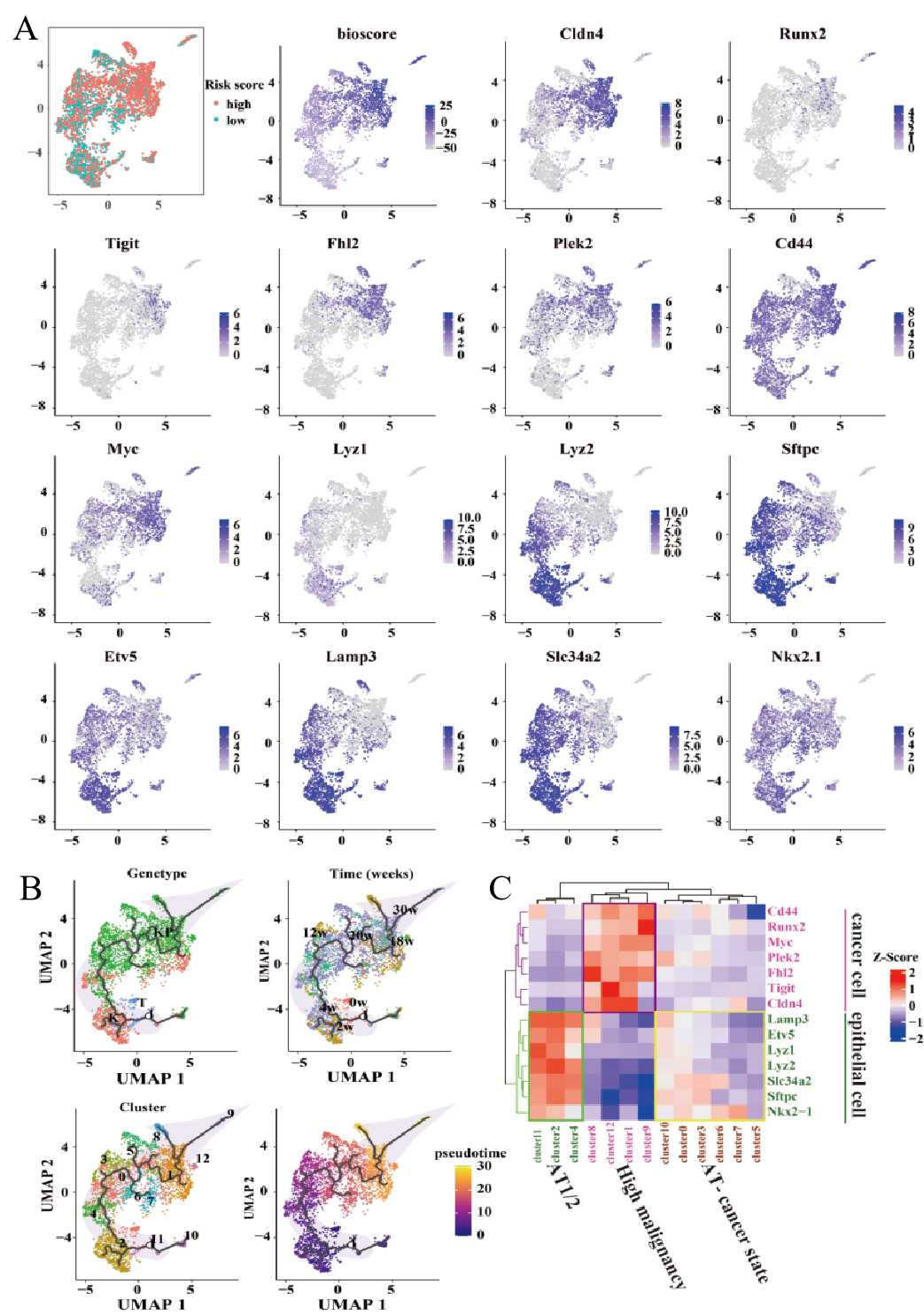


**Supplementary Figure 7.** Cell trajectory and pseudo-time analysis based on LUAD or lung epithelial marker genes. (A) Bioscore: the difference between the sum of epithelial genes in lung adenocarcinoma and the average of marker genes in lung epithelial cells. The remaining genes were derived from LUAD marker genes and lung epithelial CellMarker genes provided by CellMarker website. (B) Distribution of genotypes or experimental time. (C) Average value of lung epithelial and lung adenocarcinoma marker genes in different clusters.


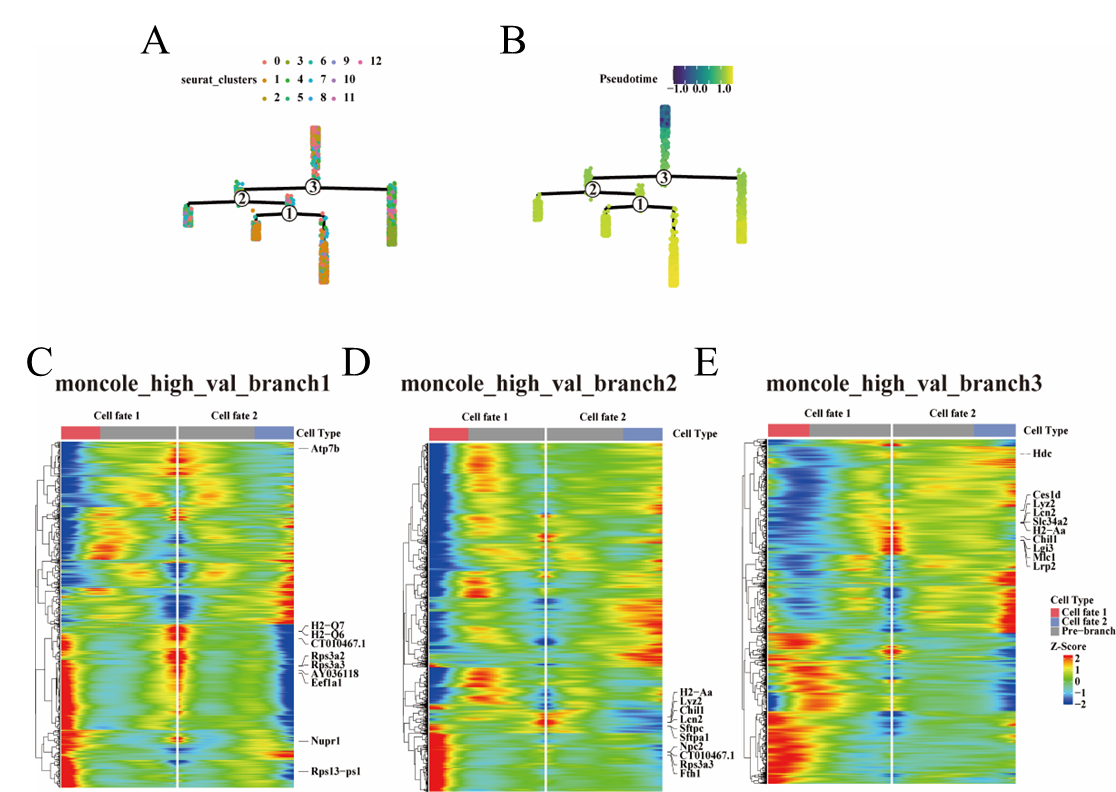


**Supplementary Figure 8.** Cell trajectory and pseudo-time analysis of branch genes. (A) Distribution of different clusters in the minimum spanning tree (different colors indicate different clusters). (B) Distribution of pseudo-time score in the minimum spanning tree (different colors represent pseudo-time score values). (C) Branch 1 related gene (FDR<10-5). (D) Branch 2 related genes (FDR<10-5). (E) Branch 3 related genes (FDR<10-5).


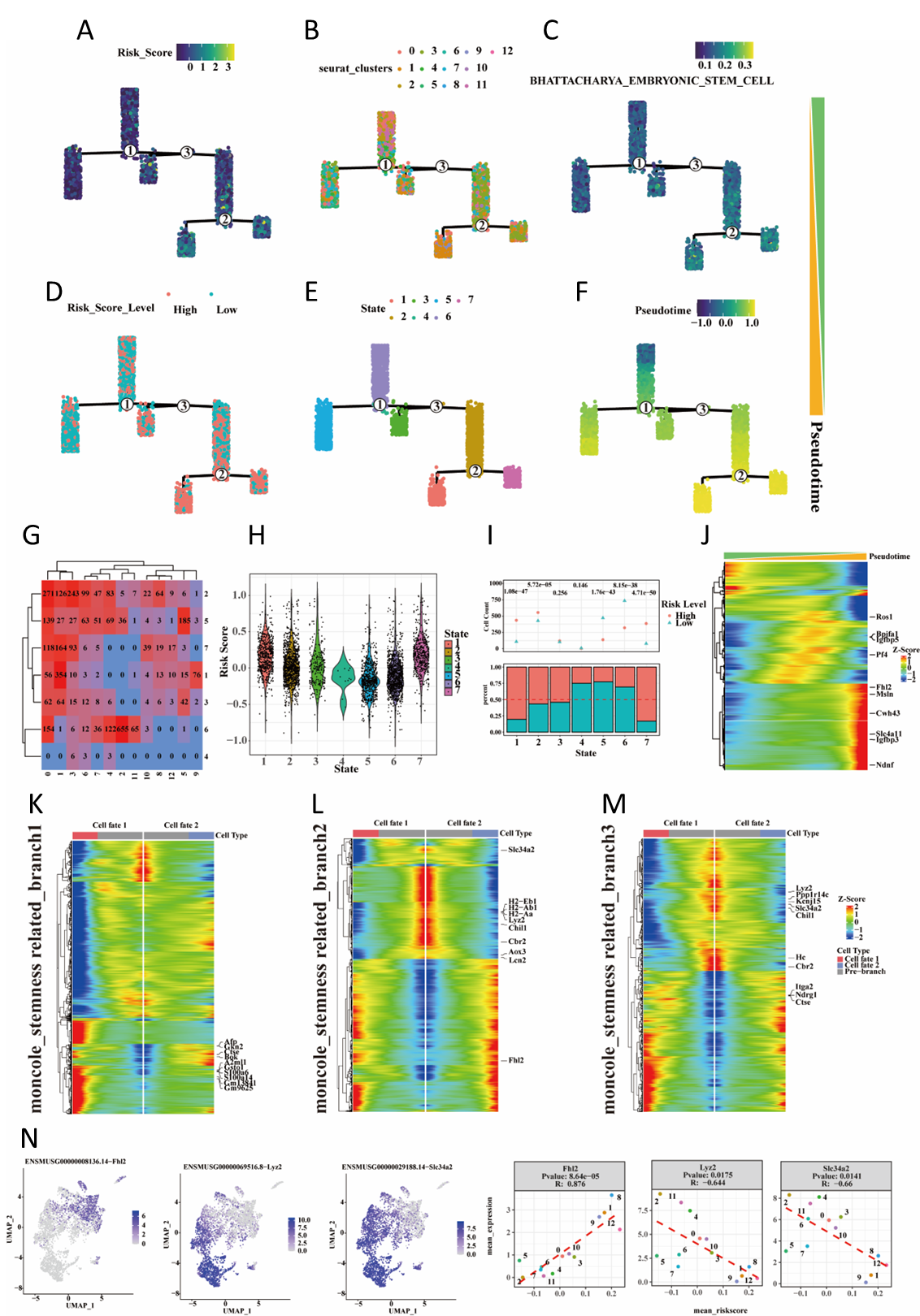


**Supplementary Figure 9.** Cell trajectory and pseudo-time analysis based on stemness related genes. (A) Distribution of cell risk score in minimum spanning Tree (different colors indicate different risk score). (B) Distribution of different clusters in the minimum spanning Tree (different colors indicate different clusters). (C) Distribution of “BHATTACHARYA_EMBRYONIC_STEM_CELL” score in the minimum spanning Tree (Color indicates the score of AUCell enrichment score). (D) Distribution of risk rating in the minimum spanning tree (red: cells with a risk score greater than -0.1, green: cells with a risk score lower than -0.1). (E) Distribution of different cell states in the minimum spanning tree (different colors indicate different cell states). (F) The distribution of different cell pseudo-time values in the minimum spanning tree (color indicates pseudo-time values). (G) The relationship between cell states and clusters in different pseudo-time series (red: high, blue: low, the number represents the number of cells in the corresponding state). (H) Cell risk values corresponding to different pseudo-time states (different colors indicate different cell states). (I) The ratio of cell risk states corresponding to different pseudo-time states (figure above: the top number represents the significance of the binomial distribution test, the red circle represents the number of cells with a risk score higher than -0.1, the green triangle represents the number of cells with a risk score lower than -0.1; Figure below: the ratio of high risk cells to low risk cells in each state, red: high risk, green: low risk, and the red dotted line indicates the value of 0.5). (J) Genes related to pseudo-time series (the genes marked on the right are the 10 genes with the strongest correlation, and different colors represent the average expression levels of different genes corresponding to different pseudo-time series). (K-M) Branch 1 related gene (FDR<10-5). Branch 2 related gene (FDR<10-5). Branch 3 related gene (FDR<10-5). (N) Distribution of pseudo-time related genes and branching genes.


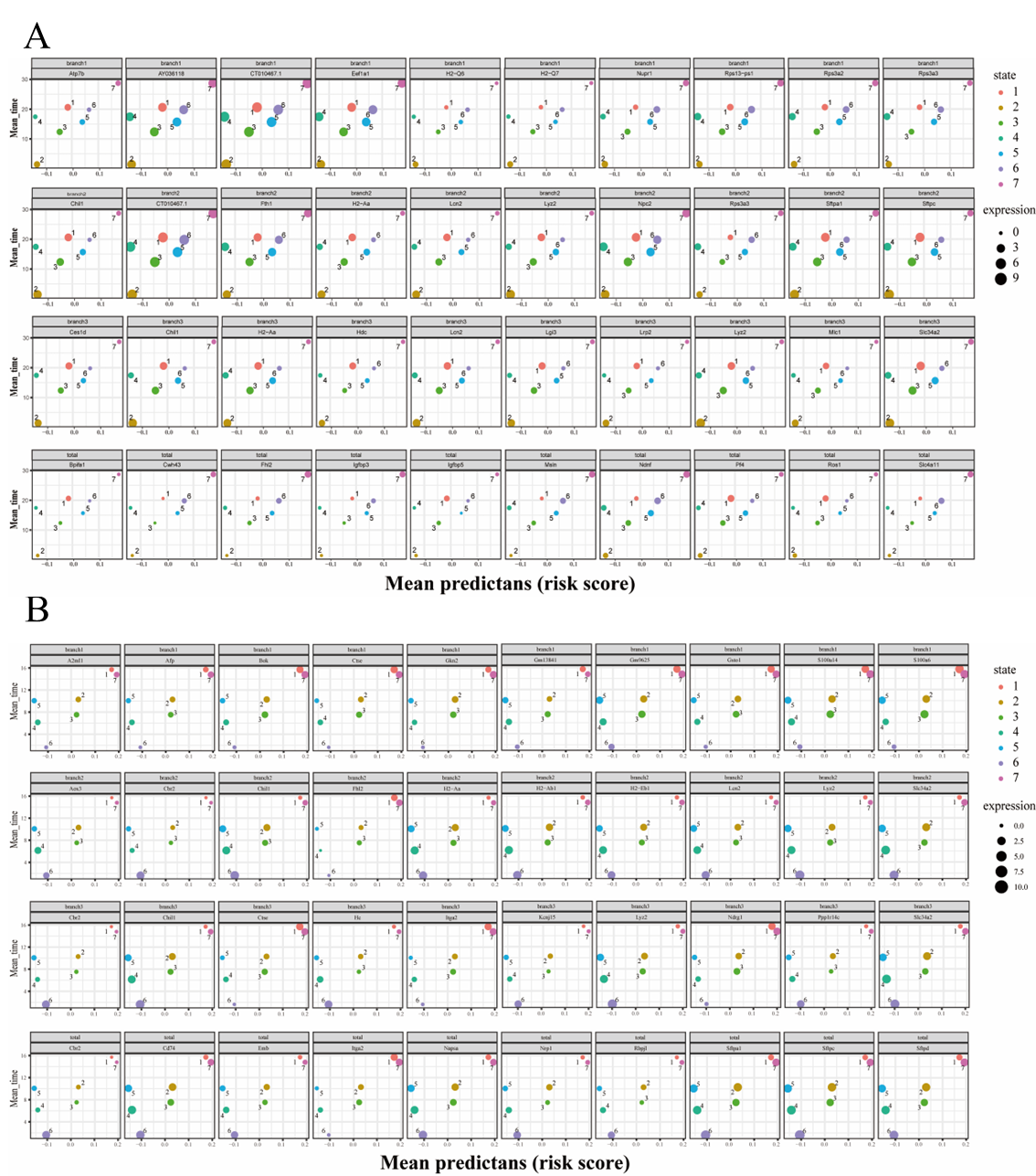


**Supplementary Figure 10.** The distribution of pseudotime related genes expression. (A) The distribution of pseudotime related high variable genes expression in each monocle state. (B) The distribution of pseudotime related stemness genes expression in each monocle state.

## Supplementary Tables

Table S1. Clinical characteristics of the LUAD patients in TCGA database.

| **TCGA** | **Risk Score** | | **X^2^** | **p** |
| --- | --- | --- | --- | --- |
|  | **High** | **Low** |  |  |
| **Age** |  | | | |
| < 60 | 76 | 67 | 1.3831 | 0.2396 |
| >= 60 | 172 | 195 |  |  |
| NA | 4 | 6 |  |  |
| **Gender** |  | | | |
| Female | 124 | 156 | 3.881 | 0.04884 |
| Male | 128 | 112 |  |  |
| **Invasion depth** |  | | | |
| T 1 | 70 | 103 | 7.0844 | 0.02895 |
| T 2/3/4 | 181 | 163 |  |  |
| T X | 1 | 2 |  |  |
| **Distant metastasis** |  | | | |
| M 0 | 170 | 178 | 3.8524 | 0.1457 |
| M 1 | 16 | 8 |  |  |
| M X | 64 | 78 |  |  |
| NA | 2 | 4 |  |  |
| **Lymph node metastasis** |  | | | |
| N 0 | 147 | 193 | 27.305 | 1.177e-06 |
| N 1/2/3 | 104 | 61 |  |  |
| N X | 1 | 13 |  |  |
| NA | 0 | 1 |  |  |
| **TNM stage** |  | | | |
| Stage Ⅰ | 115 | 171 | 19.349 | 1.089e-05 |
| Stage Ⅱ/Ⅲ/Ⅳ | 136 | 90 |  |  |
| NA | 1 | 7 |  |  |

Table S2. Clinical characteristics of the LUAD patients in GEO database.

| **GEO** | | **Risk Score** | | **X^2^** | **p** |
| --- | --- | --- | --- | --- | --- |
|  |  | **High** | **Low** |  |  |
| GSE31210 cohort | **Smoking** |  | | | |
|  | Ever-smoker | 56 | 43 | 9.3359 | 0.002247 |
|  | Never-smoker | 36 | 69 |  |  |
|  | **Gender** |  | | | |
|  | Female | 39 | 70 | 7.42 | 0.00645 |
|  | Male | 53 | 42 |  |  |
|  | **TNM Stage** |  | | | |
|  | Stage Ⅰ | 61 | 101 | 16.179 | 5.763e-05 |
|  | Stage Ⅱ | 31 | 11 |  |  |
|  | **Relapsed** |  | | | |
|  | Not Relapsed | 54 | 96 | 17.582 | 2.752e-05 |
|  | Relapsed | 38 | 16 |  |  |
|  | **Age** |  | | | |
|  | < 60 | 42 | 47 | 0.14949 | 0.699 |
|  | >= 60 | 50 | 65 |  |  |
|  | **Lymph node metastasis** |  | | | |
|  | N 0 | 40 | 54 | 7.7299 | 0.005431 |
|  | N 1 | 24 | 9 |  |  |
|  | Relapsed |  | | | |
|  | Recurrence-N | 38 | 49 | 4.9868 | 0.08263 |
|  | Recurrence-U | 2 | 1 |  |  |
|  | Recurrence-Y | 24 | 13 |  |  |
| GSE50081 cohort | **Gender** |  | | | |
|  | Female | 24 | 38 | 5.8476 | 0.1193 |
|  | Male | 40 | 25 |  |  |
|  | **Invasion depth** |  | | | |
|  | T 1 | 9 | 34 | 20.829 | 5.021e-06 |
|  | T 2/3 | 55 | 29 |  |  |
|  | **Age** |  | | | |
|  | < 60 | 9 | 10 | 0.0013853 | 0.9703 |
|  | >= 60 | 55 | 53 |  |  |
|  | **TNM Stage** |  | | | |
|  | StageⅠ | 38 | 54 | 9.7527 | 0.001791 |
|  | StageⅡ | 26 | 9 |  |  |
|  | **Smoking** |  | | | |
|  | Current | 21 | 15 | 5.8476 | 0.1193 |
|  | Ex-smoker | 28 | 28 |  |  |
|  | Never | 7 | 16 |  |  |
|  | Unable to determine | 8 | 4 |  |  |
